# Antifouling coatings can reduce algal growth while preserving coral settlement

**DOI:** 10.1038/s41598-022-19997-6

**Published:** 2022-09-24

**Authors:** Lisa K. Roepke, David Brefeld, Ulrich Soltmann, Carly J. Randall, Andrew P. Negri, Andreas Kunzmann

**Affiliations:** 1 Leibniz Centre for Tropical Marine Research, Bremen, Germany; 2 Institut Für Chemie Und Biologie Des Meeres, Carl-Von-Ossietzky Universität Oldenburg, Wilhelmshaven, Germany; 3 Gesellschaft Zur Förderung Von Medizin-, Bio- Und Umwelttechnologien E.V, Dresden, Germany; 4 Australian Institute of Marine Science, Townsville, Australia

**Keywords:** Ecology, Ecology

## Abstract

In the early stages after larval settlement, coral spat can be rapidly overgrown and outcompeted by algae, reducing overall survival for coral reef replenishment and supply for restoration programs. Here we investigated three antifouling (AF) coatings for their ability to inhibit algal fouling on coral settlement plugs, a commonly-used restoration substrate. Plugs were either fully or partially coated with the AF coatings and incubated in mesocosm systems with partial recirculation for 37 days to track fouling succession. In addition, settlement of *Acropora tenuis* larvae was measured to determine whether AF coatings were a settlement deterrent. Uncoated control plugs became heavily fouled, yielding only 4–8% bare substrate on upper surfaces after 37 days. During this period, an encapsulated dichlorooctylisothiazolinone (DCOIT)-coating was most effective in reducing fouling, yielding 61–63% bare substrate. Antiadhesive and cerium dioxide (CeO_2−x_) nanoparticle (NP) coatings were less effective, yielding 11–17% and 2% bare substrate, respectively. Average settlement of *A. tenuis* larvae on the three types of AF-coated plugs did not statistically differ from settlement on uncoated controls. However, settlement on the NP-coating was generally the highest and was significantly higher than settlement found on the antiadhesive- and DCOIT-coating. Furthermore, on plugs only partially-covered with AF coatings, larval settlement on coated NP- areas was significantly higher than settlement on coated antiadhesive- and DCOIT-areas. These results demonstrate that AF coatings can reduce fouling intensity on biologically-relevant timescales while preserving robust levels of coral settlement. This represents an important step towards reducing fine-scale competition with benthic fouling organisms in coral breeding and propagation.

## Introduction

Tropical coral reefs are under increasing pressure^1–3^ from multiple anthropogenic threats including ocean warming and acidification on a global scale and eutrophication, pollution and increased human activities in coastal areas^4–6^. An estimated 20% of reefs worldwide have been lost as a consequence of natural and anthropogenic disturbances, and a further 15% are in a critical state and likely to be lost within the next few decades^4,7^. Successful reproduction and recruitment of corals is critical for the recovery of scleractinian coral species and for increasing reef resilience^8^. High mortality after larval settlement in corals can be caused by accidental removal by grazing fish^9,10^, competition with other benthic organisms^11,12^, sedimentation^13,14^, and direct corallivory^10^. Competition with benthic algae, often triggered by excess nutrients associated with runoff is also recognized as a key threat to coral settlement and the survival of newly settled corals (spat)^15,16^.

Algal growth occurs following the primary colonization of hard benthic surfaces by microbial (bacteria, fungi, protozoa, etc.) biofilms^17^, and the settlement of corals can be either induced or inhibited by these early fouling communities, depending on the colonizing taxa^18^. Some biofilms and crustose coralline algae (CCA) are strong inducers of settlement of coral larvae^19–23^. However, the interaction between corals and other fouling organisms, such as filamentous algae, can be detrimental to larval settlement and to the survival of coral spat, juveniles and the success of adult corals^24–26^. Fouling can impact coral spat through competition for space and direct overgrowth by microorganisms or motile propagules of other macroorganisms^27^. Furthermore, the microtopography provided by fouling may provide a refuge for microbial pathogens^28^ or corallivorous invertebrates. Allelopathy^29^, shading^27^, alteration of fine-scale water movement, and other microenvironmental changes induced by fouling algae may also impede coral survival or fitness of coral spat^30^.

Declining coral cover and health, and increasing environmental pressures on tropical coral reefs, have motivated the development of novel restoration techniques and the establishment of restoration programs around the globe^31–33^. A key strategy for large-scale reef restoration is to deliver captured or cultivated coral larvae onto the reef, either directly^34–36^ or on deployment devices^37–39^. The benefits of using sexually produced coral propagules for this purpose include improvements in genetic diversity, scalability and cost^38,40,41^, as well as retention of species diversity and community composition^41^. Deploying spat onto reefs using devices also, at least temporarily, overcomes challenges associated with the settlement process, including a lack of available substrate or settlement cues^42–44^, the presence of settlement inhibitors^45–47^, and direct competition with surrounding benthos. The application of antifouling (AF) technologies onto deployment devices to increase the survival of sexually propagated corals is a potential strategy to support coral spat and recruit survival over the first vulnerable months^48^.

AF paints containing biocides that inhibit the establishment of algal and invertebrate communities have been applied on ship hulls to increase efficacy since the mid nineteenth century^49^. High concentrations of tributyltin (TBT) and copper-based AF paints that can contaminate coral reefs following groundings have previously been considered a threat to coral larval settlement^50^ and spat survival^51^. However, the banning of TBT and further regulation of copper in AF paints has led to the development of alternative formulations that either contain no biocides or less toxic biocides that rapidly degrade, reviewed in Almeida et al.^49^. One of the most widely applied AF coatings, Sea-Nine 211™ (Röhm & Haas; Philadelphia, PA, USA^52^), includes an active biocidal ingredient, known as dichlorooctylisothiazolinone (DCOIT, Kathon 930, C-9 or DCOI). DCOIT can diffuse across the cell walls and membranes, binding to proteins and enzymes, blocking activity in biofilms, target and non-target organisms^53^. The half-life of DCOIT depends on environmental conditions, ranging from less than one day to 10 days or more, and can be influenced by several biotic and abiotic factors, such as microorganisms, photolysis, hydrolysis, pH and salinity^54^. Toxic effects have been observed across a diversity of organisms including bacteria, fungi, algae, bivalves, echinoderms, ascidians, fish, copepods, decapods, and soft corals^55–62^. Although the toxicity mechanism of DCOIT is not fully understood, embryotoxicity, immunosuppression, oxidative stress, reproductive and endocrine disruption in marine organisms have been reported^63^. However, some studies have shown effective AF properties of DCOIT encapsulated in silica nanocapsules which can decrease the toxicity towards non-target organisms^64,65^. Yet, no study to date has investigated the effects of DCOIT on hard corals, or encapsulated in a sol–gel.

Non-biocidal antifoulants include silicon-based sol–gel coatings, which are produced by a wet-chemical technique used for the fabrication of metal oxides. The sol (or solution) gradually forms a gel-like network containing both a liquid and a solid phase. The antiadhesive surface properties of these coatings can prevent the attachment of fouling organisms and facilitate their release at higher water velocities. The antiadhesive effect is achieved through low surface energies (with weak molecular attraction), amphiphilic properties (both hydrophilic and hydrophobic properties) and a precisely tuned surface roughness tuned to inhibit primary fouling^66^. Other recently developed non-biocidal AFs are based on nanoparticles, which disrupt bacterial cell-to-cell communication (i.e., quorum sensing) to inhibit the formation of biofilms and mitigate or delay later colonization by algae^67^. One example includes cerium (Ce^4+^/Ce^4+^)-modified sites across the high surface area of NPs, which enhance the catalytic oxidation of halides, resulting in the formation of biocidal compounds which combat biofilm formation or the formation of signaling molecules involved in intracellular communication^68–70^. Laboratory and field tests with paint formulations containing 2 wt% CeO_2−x_ NPs showed a higher reduction in biofouling than Cu_2_O, the most common biocidal ingredient in AF paints^68,70^.

Only a single study has attempted to reduce fouling on surfaces to improve coral spat survival^48^. That study demonstrated how the application of two different paraffin waxes in close proximity to early coral spat significantly reduced fouling and improved survival, and no differences in coral settlement between wax treatments and uncoated controls were found. Further research is needed on the potential for recently-developed AF coatings to mitigate competition from biofouling and increase the likelihood of coral survival to size-escape thresholds (sensu Doropoulos et al.)^71^. This would ultimately improve the feasibility of sexual propagation techniques for reef-restoration programs.

In this study, we manufactured and tested the efficacy of three distinct AF coatings: an antiadhesive, a cerium dioxide nanoparticle (NP) coating, and an encapsulated DCOIT-coating. In addition, we investigated the settlement of *A. tenuis* coral larvae on fully-coated (FC) and partially-coated (PC) settlement surfaces (“coral plugs” or “plugs”). Our objectives were to (1) explore the AF efficacy to reduce biofouling, (2) explore the fouling community (CCA, green/brown algae, bare substrate) composition and (3) assess coral-settlement preferences on coated and uncoated areas of pre-conditioned plugs. Our ultimate goal was to test whether the coatings reduced algal growth without inhibiting larval settlement adjacent to the coatings.

## Materials and methods

### Coral plug preparation and coating design

**Figure 1:**
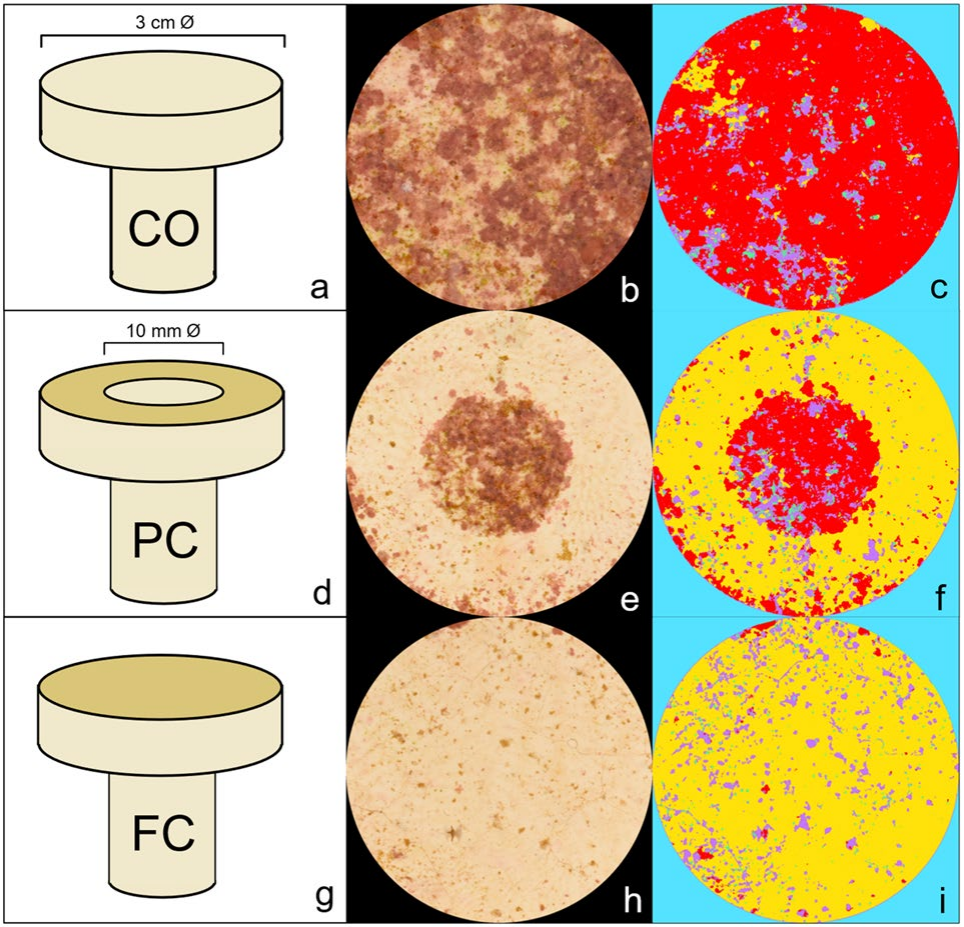
Uncoated Control (CO; **a)**, partially-coated (PC; **d**), and fully-coated (FC; **g**) plugs, cropped images of CO (**b**), PC (**e**), and FC (**h**) DCOIT plugs (after 37 days in the aquaria), and the segmentation of fouling classes on the same CO (**c**), PC (**f**), and FC (**i**) DCOIT plugs, using machine learning-based image classification with the Trainable Weka Segmentation (TWS) in ImageJ. Each fouling class was segmented using a specific color (**c**,**f**,**i**): red (CCA), violet (brown algae), green (green algae), yellow (bare substrate), blue (background).

Coral plugs (top disc diameter: 3 cm) made from white Portland cement were purchased (AquaPerfekt, Germany; Dyckerhoff WEISS CEM 1) and the layer of sand (Raunheimer quartz sand) on top of the surfaces was removed with a diamond saw blade on a circular saw. A smooth finish was achieved by sanding and polishing the plug’s surfaces on very fine wet sandpaper (STARCKE® (SiC) waterproof Matador 220, 800, 2500). The uniformly smooth top surfaces were then coated with three different AF coatings following the methods described below. For each AF coating, half of the plug replicates were fully-coated (FC) and the other half were partially-coated (PC) (Fig. 1), leaving a circular control area in the center of each plug uncoated (Fig. 1). A circular sticker (12 mm Ø) was applied to the plug and prevented the coating in an area of approximately 10 mm Ø, since the sol spread out slightly underneath the sticker (approximately 88% coated and 12% uncoated). Control plugs were not coated. The purpose of including FC plugs, was to explore the antifouling efficacy, fouling community composition and coral larval settlement preferences among the AF treatments. PC plugs were used to further explore fine-scale antifouling efficacy, fouling community composition and coral larval settlement preferences, when offered the choice to settle on an uncoated or coated area.

### AF coating manufacturing

#### CeO_2−x_ nanoparticle coating

Cerium oxide nanoparticles (NPs) were manufactured according to Ji et al.^72^ and the Supporting Information in Herget et al.^73^ with minor adjustments as described below.

The process involved: (i) the production of nanoparticles, (ii) the catalytic activity testing, (iii) the production of coating solution A which included tetraethoxysilane (TEOS) and (iiii) the production of coating solution B which included TEOS and 3-glycidyloxypropyltriethoxysilane (GLYEO) (all technical details can be found in the Supporting Information). The plugs were coated 3 times, with a base coat and two overcoats. First, plugs were dip-coated with coating solution A, followed by a second and third coating applied by a soaked felt (textile) with solution B. The combination of an initial dip coating using a SiO_2_-sol with CeO_2−x_ and the subsequent overcoats using an epoxy-modified sol with CeO_2−x_ gave good results in terms of adhesion and uniformity of the coatings. On the unmodified basecoat, the epoxy-modified sols showed good coating adhesion, with fewer tendencies to crack. For the overcoats, a more homogeneous distribution of the CeO_2−x_ particles was achieved by using a felt for the coating application. Test coatings on Poly (methyl methacrylate) (PMMA) tiles (16 cm^2^) revealed a coating weight of 14.4 mg/16 cm^2^ (0.9 mg/cm^2^). The final concentration of ceria NPs in the coating solution was 35.7 wt%. Thus, the final surface density of ceria NPs was approximately 0.32 mg/cm^2^.

#### Antiadhesive coating

Following and adapting the methods by Sokolova et al.^74^ and Detty et al.^66^ a silica-based sol–gel was produced from *n*-octadecyl-trimethoxysilane (C18, 0.675 g, CAS 3069-42-9, abcr GmbH, Karlsruhe), tridecafluoro-1,1,2,2-tetrahydrooctyltriethoxysilane (TDF, 3.675 g, CAS 51851-37-7, abcr GmbH, Karlsruhe), *n*-octyltriethoxysilane (C8, 22.4 g, CAS 2943-75-1, abcr GmbH, Karlsruhe), and tetraethylorthosilicate (TEOS, 18.75 g, CAS 78-10-4 abcr GmbH, Karlsruhe) in a molar ratio of 1:4:45:50. The modified silica alkoxides were mixed with 59.5 mL of ethanol and 11.4 mL of HCl (0.1 M) and stirred for 24 h at room temperature for sol formation. Prior to coating the surface, the plugs were cleaned with ethanol and their surfaces were activated by microwave plasma (working pressure 10 Pa in air, activation time 2.5 min., Creaetch 250 Plasma MV, Creavac GmbH) to ensure good coating adhesion. Coatings were applied by the dip-coating technique and cured at 100 °C for one hour.

#### DCOIT coating

Dichlorooctylisothiazolinone (DCOIT; C_11_H_17_Cl_2_NOS; TCI America; CAS RN 64359-81-5; Product number D4157) was dissolved in ethanol and attached to hydrophobic pyrogenic silicic acid, each batch containing 0.5 g DCOIT in 2 mL ethanol and 0.1 g hydrophobisated silicic acid powder. After gentle stirring, the mixture was incubated overnight at room temperature, loosely covered, to allow ethanol to evaporate. Subsequently, the DCOIT loaded silicic acid powder was incorporated in two different sol-gels: 1. an aqueous SiO_2_-sol made from TEOS and 3-glycidyloxypropyl-triethoxysilane (9 mL TEOS, 1 mL 3-glycidyloxypropyl-triethoxysilane, 60 mL H_2_O and 30 mL 0.01 M HCl were stirred for 20 h) and 2. a hydrophobic SiO_2_-sol (same antiadhesive coating as in Sect. “Antiadhesive coating”). Each SiO_2_-sol with 20 mL volume contained 0.1 g of DCOIT loaded silicic acid powder. After mixture, the dispersions were sonicated (UP100H, Hielscher Ultrasonics GmbH, Teltow, Germany) for 3 min, followed by a 5 min cooling period to prevent heating of the dispersions, followed by another 3 min sonication. Prior to coating the surface, the plugs were cleaned with ethanol and their surfaces were activated in microwave plasma (working pressure 10 Pa in air, activation time 2.5 min., Creaetch 250 Plasma MV, Creavac GmbH). Plug surfaces were dip-coated in the hydrophilic sol first, then dried and tempered at 100 °C for one hour. Afterwards, the second hydrophobic sol was applied by dip-coating, then dried and tempered at 100 °C for an hour. To estimate the embedded DCOIT concentration per area, DCOIT was extracted from the coatings with ethanol. The concentration was measured photometrically at 280 nm. A DCOIT concentration of 3.2 mg/cm^2^ was determined.

### Fouling experiment

#### Experimental setup

Plugs were kept in three replicate outdoor holding mesocosm aquaria (280 L) located in the quarantine area of the National Sea Simulator (SeaSim) of the Australian Institute of Marine Science (AIMS) in Townsville, Australia. Each tank contained 15 replicate plugs per treatment (15 plugs × 7 treatments including the control treatment). In total, 315 plugs were tested (3 tanks × 105 plugs). Each aquarium was independent of the others, with a separate recirculation system and an independent, temperature-controlled sump. Fresh filtered seawater was circulated into each experimental tank at a rate of three full water exchanges (turnovers) per day. Temperature was maintained at 26 °C and measured every 60 min. (HOBO Pendant® Temperature/Light 64 K Data Logger, Onset Computer Corporation, Bourne, MA) and salinity was kept in the range of 34.5 to 35.5 PSU. Water circulation in the tanks was maintained by Maxspect Gyre 200 series pumps (constant speed mode, 20% force). Tanks received natural sunlight attenuated by ~ 70% with shade cloth: maximum Photosynthetically Active Radiation (PAR) light intensity was 250 μmol photons m^−2^ s^−1^. PVC plug trays (custom-fabricated: 40 × 20 × 5 cm (length x width x height), holes (1.2 cm Ø) for coral plugs arranged in rows A-E with 10 holes per row) were used to maintain the coral plugs horizontally within the tanks. Tray positions were swapped circularly on a weekly basis to minimize within-tank effects (i.e., unequal shading or local water flow) on fouling across the plugs. To ‘seed’ the plugs with a biofilm, approximately 3 kg of live rock (comprising reef rubble with typical fouling communities incl. CCA) was added to each tank 3 weeks before plugs were introduced.

#### Fouling quantification in ImageJ

To monitor and quantify the fouling intensity on the plugs, plug surfaces were photographed at regular intervals (after 9, 23 and 37 days) using a Nikon D810 with a Nikon AF-S 60 mm f/2.8 G Micro ED Lens outfitted with four Ikelite DS160 strobes mounted on a trolley with standardized distance and angle to each plug surface. The monitoring intervals were chosen based on other studies with similar coatings^66,68^. Six plugs were photographed at once. All image analyses were performed in ImageJ version 1.53c^75,76^. To obtain images of single plugs, each plug was cropped out of the original image using a standardized circular region of interest (ROI; Fig. 1). The ROI was slightly smaller (2.5 cm Ø) than the plug surface’s diameter, to easily accommodate all plugs. Subsequently, the Trainable Weka Segmentation (TWS) plugin in the Fiji distribution of the image processing program ImageJ^77^ was used to segment images into different classes by the use of machine-learning based image classification models. The analysis was adapted from the “Trainable Weka Segmentation User Manual” published as Supplementary data in Arganda-Carreras et al.^78^, but other studies have previously applied similar approaches to quantify biofouling with the TWS^79–82^. More recently, Macadam et al.^83^ successfully used a similar machine-learning tool to measure coral spat survival, size, and color. In this study, each plug image was segmented into 5 different classes: crustose coralline algae (CCA), green algae, brown algae, bare substrate and background. Green and brown algae were subsequently combined into a single category “green/brown algae” for statistical analysis, as the TWS classification of green and brown algae did not consistently achieve the same accuracy as for the category CCA and bare substrate. The initial model training stack was created using nine randomly selected images, one from each tank and monitoring period (3 tanks × 3 monitoring periods = 9 plug images). After loading the image stack into the TWS plugin, a minimum of three ROIs were selected for each fouling class on every image and the base model was trained using the default image features. By selecting just one image feature at a time and re-training the model, the most promising image features were chosen based on their minimization of the “out of bag error” (OOBE). The OOBE is a recommended option in TWS to measure the prediction error of the machine learning models by utilizing bootstrap aggregating methods. A lower OOBE indicates a better model performance. Features with no substantial reduction of the OOBE were excluded from the model. The minimization of the OOBE by each of the chosen image features was confirmed by excluding one feature at a time and re-training the model. By removing features, which did not minimize the OOBE in this step-wise backward-selection, a set of five features (variance, minimum, maximum, structure & neighbors) were chosen for further analyses. If the initial model segmented the image satisfactorily into the chosen fouling classes, the classifier was applied to all images and its accuracy was validated visually. The initial model was optimized using four more training stacks of formerly mis-segmented images (mis-segmentations of CCA, green/brown algae and bare substrate after visual validation). In total, 40 images of plugs were used for model training. Classes were balanced and the default classifier with 200 boosted regression trees, each considering 2 random features, was used. The OOBE for the final model was 0.591%. Pixels of areas with overlapping fouling classes (e.g. brown algae overgrowing CCA) were classified as the “more dominant” class, resulting in each pixel being assigned only to a single class. Corresponding areas were included during model training. After classification, the fraction of each class from the total plug surface was measured by using the particle analyzer in ImageJ. The segmented 8-bit images include a look-up table (LUT) in which each class is specified with a specific color (Fig. 1). Using the thresholding tool, segments of one class were extracted and masks were produced. Using the particle analyzer, the percentage of each area belonging to one class was calculated and the results were exported to Microsoft Excel 2019. The classification of the PC plugs was performed for the whole surface area (coated and uncoated). Afterwards, each image was segmented into coated and uncoated regions and the remaining analyses were conducted as for the FC plugs. The cropped images, as well as the ROIs, segmented images, and the final model and training data can be accessed from a on-line repository here: https://doi.org/10.25845/ws1n-ah16.

### Coral settlement experiment

#### Coral collection and larval husbandry

Fragments of gravid colonies (25–40 cm diameter) of the scleractinian coral *Acropora tenuis* (Dana, 1846) were collected from Davies Reef (18° 49′35.70″ S 147° 37′36.46″ E; 4 m depth) on December 12, 2019 under permit G12/35,236.1 issued by the Great Barrier Reef Marine Park Authority. Colonies were transported to the SeaSim and maintained in 1700 L semi-recirculating holding tanks until spawning. Temperatures were held at 26–27 °C, to match the seawater temperature at the collection site. Spawning occurred on 17th December 2019 and the coral gametes from seven parental colonies were collected and fertilized. Symbiont-free larval cultures were maintained at densities < 500 larvae per L, indoors, in 70 L flow-through rearing tanks (1.5 turnovers per day) supplied with 1 μm filtered seawater at 27 °C^84,85^. Larvae remained in those conditions until the experiment commenced.

#### Experimental setup

*A. tenuis* larvae (each 800–1000 µm in length) were competent to undergo attachment and metamorphosis after 5 days, as determined by routine settlement assays in the laboratory^19^. Settlement was defined here as the change in life stage from “free-swimming” or “loosely-attached, elongated” larvae to a squat, firmly attached and disc-shaped form, with pronounced flattening of the oral–aboral axis and with septal mesenteries radiating from the central mouth region^19^. The same plugs that were photographed to quantify fouling intensity (described above), were subsequently used to test larval settlement success in this experiment. Due to handling time restrictions (pipetting larvae and counting coral spat), the number of experimental plugs for settlement was reduced to 30 plugs per treatment (chosen haphazardly, 7 treatments: control, FC NPs, FC antiadhesive, FC DCOIT, PC NPs, PC antiadhesive, PC DCOIT), for a total of 210 plugs. After snapping off the stem of each plug, the plugs were isolated from each other by placing them in individual 200 mL glass jars, haphazardly interspersed, and filled with 150 mL of 1 μm filtered seawater. Prior to the start of the experiment, clear glass jars (7 cm Ø, 11 cm height) were cleaned with 20 mL acetone each, left to dry under a drying cabinet, and then rinsed in filtered seawater. To prevent larvae from attaching and undergoing metamorphosis on the side or underside of the plug, plugs were gently set into a thin layer (about 2 cm depth) of fine glass beads (1 mm Ø) placed at the bottom of each jar. Prior to filling the glass jars, the newly purchased beads were rinsed in freshwater twice and then spread as a thin layer (0.5 cm depth) on aluminium foil to dry in an oven at 50 °C for 5 h. The glass jars and glass beads were generic (no brand). 15 randomly selected 11-day-old larvae were pipetted gently into each jar. No settlement inducers were added, because each fouled plug surface contained a biofilm that included crustose coralline algae (CCA). Larvae were incubated under artificial lighting (Hydra FiftyTwo HD LED, Aquaria Illumination, Allentown, USA) at an intensity of approximately 40 µmol photons m^−2^ s^−1^ (12:12 h light:dark cycle) and at a constant water temperature of 26.7 °C. Settlement was assessed after 24 h under a stereo microscope and was considered normal if a larva had undergone attachment to the plug surface and metamorphosis into a polyp, as described above. All other larvae (swimming, loosely attached, dead and partially disintegrated) were counted as not settled.

### Statistical analysis

All statistical analyses were performed in R version 4.2.0^86^ and the data were manipulated and visualized using packages of the ‘tidyverse’^87^.

Fouling intensity on the FC and PC plugs was measured as percent coverage by fouling class (CCA, green/brown algae, bare substrate). To investigate differences in fouling intensity after 37 days (end of experiment) between AF treatments, linear mixed models (LMM) were fitted using the package 'nlme'^88^. The last timepoint of the analysis shows the overall efficacy of the AF coatings and was therefore considered to be the most informative timepoint from a biological and ecological standpoint as less algae-coral competition is hypothesized with less algae fouling. On the FC plugs, fouling coverage was modeled as a function of fouling class and AF treatment. The fouling coverage on the PC plugs was modeled as a function of the area (coated vs. uncoated), fouling class and AF treatment. Both models used a square-root transformation of the response to stabilize heteroscedasticity of the residuals, and included ‘tank’ as a random effect. Furthermore, the fouling classes were corrected for unequal variances using the 'varIdent' function in the weights argument. Model fit was visually assessed using standard diagnostic plots of the normalized residuals. ANOVA tables (type II tests) were computed for both models using the 'car' package^89^ in order to identify statistically significant fixed effects and their interactions. When significant, post-hoc tests for pairwise comparisons between treatments were performed using the ‘emmeans’ package^90^. On the FC plugs, differences in fouling coverage between treatments were assessed within each fouling class. On the PC plugs, differences in fouling coverage were investigated between the coated and uncoated area, for each treatment within fouling classes. The p-value was adjusted for multiple comparisons with the Tukey method. The presented estimated marginal means were back-transformed from the square-root to the response scale for interpretation (Supplementary Figs. S1 and S2). The estimated contrasts are provided in the Supplementary Information.

Settlement was expressed as settler (spat) density (settlers/cm^2^). On the FC plugs, the settler density was calculated by dividing the total number of settlers on each plug by the total surface area (7.069 cm^2^). On the PC plugs, the settler density was calculated by dividing the number of settlers on the uncoated and coated area of each plug by the respective surface area (uncoated: 6.283 cm^2^; coated: 0.785 cm^2^). To investigate differences in settler density among treatments, LMMs were fitted. On the FC plugs, the settler density was estimated as a function of treatment using the package ‘glmmTMB’^91^. To account for the correlation of observations from the same tank, “tank” was added as a random factor. On the PC plugs, settler density was modeled as a function of treatment and area (uncoated/coated) using the package 'nlme'^88^. “Tank” again was used as a random factor and all treatment-area-fouling class combinations were corrected for unequal variances using the 'varIdent' function in the weights argument. The response variables (settler density) of both models were square-root transformed to stabilize heterogeneity in the residuals and improve model fit. Satisfaction of model assumptions was checked by using standard R model diagnostic plots. Plots were based on 'response' residuals on the FC plugs and on 'normalized' residuals on the PC plugs. ANOVA tables (type II tests) for both models were computed again using the 'car' package^89^. If significant, pairwise post-hoc tests were performed using the ‘emmeans’ package^90^ for all group combinations. P-values were adjusted for multiple comparisons using the Tukey method. Tests were performed on the square-root and back-transformed for visualization (Figs. 4, 5). Coefficients remained on the square-root.

## Results

### Biofouling

#### Biofouling: fully-coated plugs

**Figure 2:**
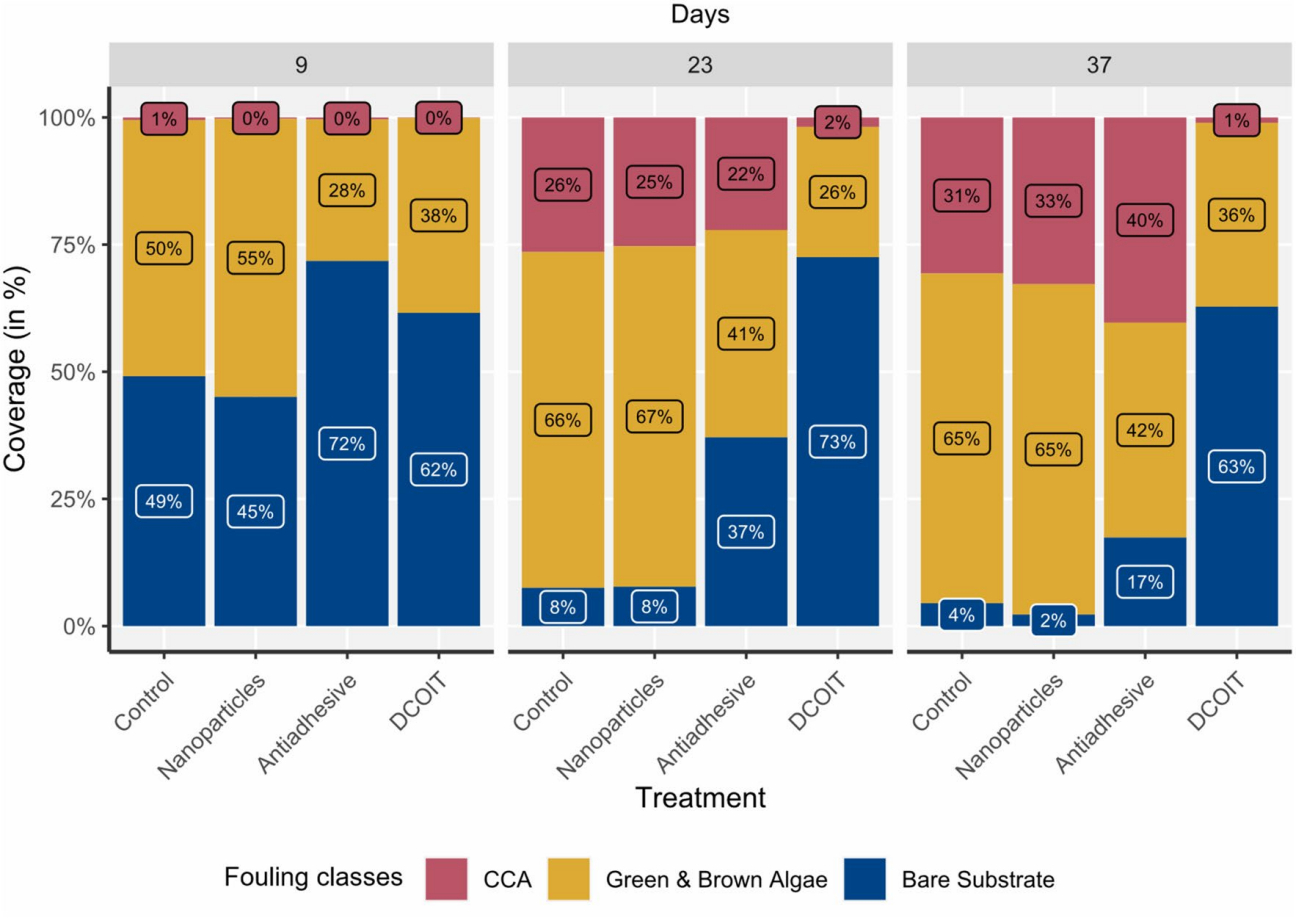
Fully-coated (FC) plugs (Nanoparticles, Antiadhesive, DCOIT) and uncoated Control plugs (n = 45 plugs per treatment) were incubated in semi-recirculating seawater systems prior to coral settlement trials to examine the mean coverage (%) of fouling classes (CCA, Green/brown algae, Bare substrate) over time (after 9, 23, 37 days). Note that bare substrate coverage at experimental start (day 0) was 100% (Supplementary Table S1).

Settlement plugs became progressively colonized with green/brown algae and crustose coralline algae (CCA) over the course of the experiment, and rapid fouling was evident after 9 days on all surfaces. Between 9 and 23 days, all plugs shifted towards higher fouling cover, except for DCOIT-coated plugs (Fig. 2; Supplementary Table S1). Antifouling treatments had a statistically significant effect on the fouling community composition after 37 days (LMM: χ^2^ (6) = 504.29, p < 0.001). This analysis focused on comparing treatments withing fouling classes, while the “full” pairwise results including treatment-fouling class interactions are shared in the Supplementary Information (Table S4). After 37 days, bare substrate was significantly different (p < 0.001) among all treatments, except between the NP treatment and control. Bare substrate on the DCOIT-coated plugs averaged 62.8 ± 3.3% (mean ± SE; Supplementary Table S1) and was significantly higher (p < 0.001) than average bare substrate on the antiadhesive-coated plugs (17.4 ± 2.2%; Fig. 2 and Supplementary Fig. S1; Supplementary Tables S1 and S3) and all other plugs. Bare substrate on the antiadhesive-coated plugs was significantly higher (p < 0.001) than average bare substrate on the NP-coated (2.3 ± 0.4%) and control plugs (4.5 ± 0.6%; Fig. 2 and Supplementary Fig. S1; Supplementary Tables S1 and S3). Green/brown algae were the most dominant fouling class in all treatments throughout the experiment (average of 52.1% across all treatments by day 37). However, after 37 days, average green/brown algae coverage on the DCOIT treatment (36.2% ± 3.4%) and antiadhesive treatment (42.3% ± 4.5%) was significantly lower than on the nanoparticle treatment (65% ± 5.3%) and the control (64.8% ± 5.1%; Fig. 2 and Supplementary Fig. S1; Supplementary Tables S1 and S3). On control plugs, CCA cover increased from 0.5% to 30.7% between days 9 and 37 (Fig. 2; Supplementary Table S1). CCA coverages on the nanoparticle-coated and control plugs were very similar after 9, 23 and 37 days (Fig. 2). After 37 days, only 1.1 ± 0.3% CCA cover was measured on the DCOIT treatment, significantly less CCA (p < 0.001) as compared to all other treatments and the control. There were no differences in CCA coverage among the NP, antiadhesive and control treatments (Fig. 2 and Supplementary Fig. S1; Supplementary Tables S1 and S3).

#### Biofouling: partially-coated plugs

**Figure 3:**
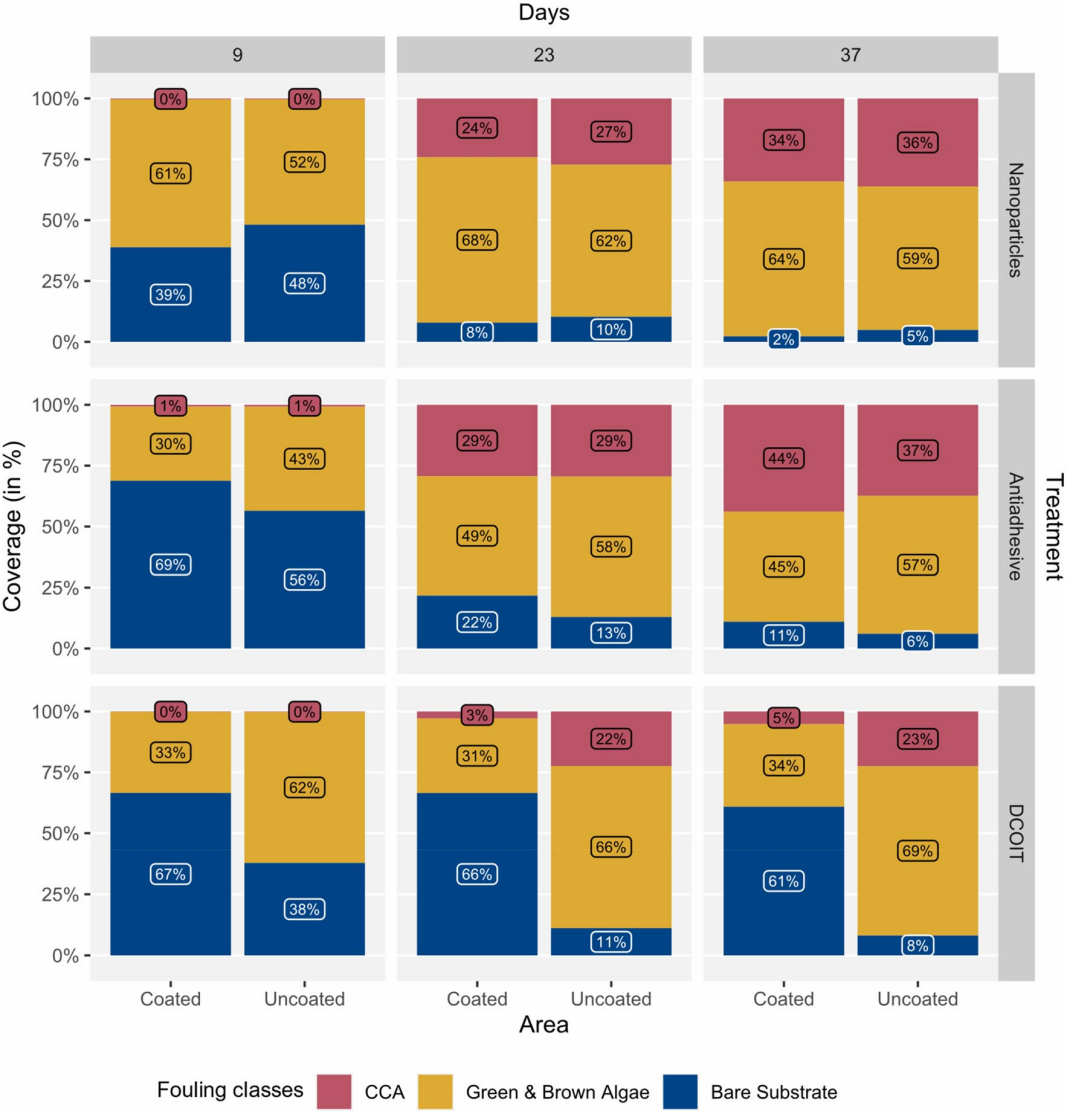
Partially-coated (PC) plugs (Nanoparticles, Antiadhesive, DCOIT) with coated and uncoated areas (n = 45 plugs per treatment) were incubated in semi-recirculating seawater systems prior to coral settlement trials to examine the mean coverage (%) of fouling classes (CCA, Green/brown algae, Bare substrate) over time (after 9, 23,37 days). Note that bare substrate coverage at experimental start (day 0) was 100% (Supplementary Table S5).

Fouling class coverages on the coated and uncoated control areas of the PC plugs were generally similar to fouling on the FC and uncoated control plugs, respectively (Fig. 2) throughout the experiment. There was a significant three-way interaction among AF treatment, area (coated vs. uncoated) and fouling classes after 37 days (LMM: χ^2^ (4) = 167.204, p < 0.001), suggesting that the differences between coated and uncoated areas were not consistent for all treatments and fouling classes. The “full” pairwise results including area-treatment-fouling class interactions are shared in the Supplementary Information (Table S8). After 23 and 37 days, the uncoated areas of the antiadhesive-coated and DCOIT-coated treatments were similar to the control plugs, whereas the coated areas of these treatments had more bare substrate (Fig. 3; Supplementary Table S5). There were significant differences in bare substrate coverage between coated and uncoated areas across all AF treatments, but only DCOIT significantly reduced CCA and green/brown algae coverage (Fig. 3; Supplementary Fig. S2 and Supplementary Table S7). Bare substrate averaged 60.9 ± 3.3% (mean ± SE) on the coated areas and was significantly higher (p < 0.001) than bare substrate on the uncoated areas (8.1 ± 1.2%) of the PC DCOIT plugs (Fig. 3; Supplementary Fig. S2 and Supplementary Tables S5 and S7). CCA were significantly more abundant (22.5 ± 4%; p = 0.005) on the uncoated areas than on the coated areas (5.2 ± 1.3%) of the PC DCOIT plugs. Green/brown algae were dominant (p < 0.001) on the uncoated areas in contrast to the coated areas (33.9 ± 3.3%) of the PC DCOIT plugs. Bare substrate on the PC antiadhesive plugs differed significantly between coated and uncoated areas (p = 0.008), with more bare substrate on the coated areas. In contrast, significantly more bare substrate was found on the uncoated areas of the nanoparticle treatment (p = 0.049) (Fig. 3; Supplementary Fig. S2 and Supplementary Tables S5 and S7).

### Settlement

#### Settlement: fully-coated plugs

**Figure 4:**
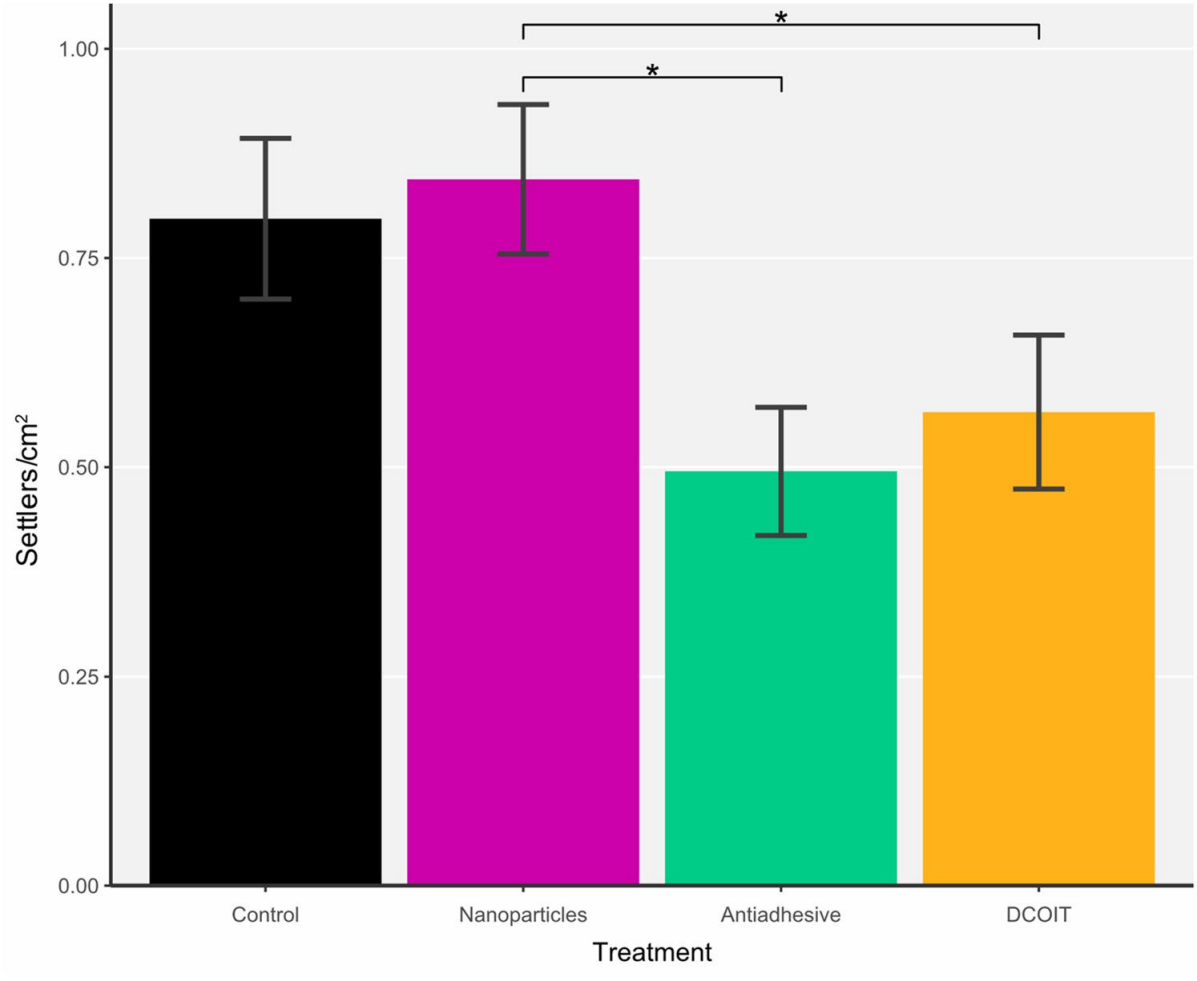
Pre-conditioned fully-coated (FC) plugs (Nanoparticles, Antiadhesive, DCOIT) and uncoated Control plugs (n = 30 plugs per treatment) were tested for mean coral larval settlement (settlers per cm^2^) in individual glass jars (one plug with 15 larvae per jar). Error bars represent SEM. Asterisks indicate statistically significant differences based on pairwise post-hoc tests with least-squares means (Supplementary Tables S9 and S11; *p < 0.05).

The average settlement success on control plugs was 0.8 ± 0.1 settlers/cm^2^ (mean ± SE), typical of settlement on fouled plugs^82^. On the fully-coated (FC) plugs, there was a statistically significant effect of treatment on larval settlement (LMM: χ^2^(3) = 15.559, p < 0.01). *A. tenuis* settlement was highest on the NP-coated plugs with an average of 0.8 ± 0.1 settlers/cm^2^ and similarly high with the control plugs, while the lowest settlement was found on the antiadhesive-coated plugs (0.5 ± 0.1 settlers/cm^2^) (Fig. 4; Supplementary Tables S9 and S11). Coral larval settlement on the DCOIT-coated plugs (0.6 ± 0.1 settlers/cm^2^) was higher than on the antiadhesive-coated plugs, and did statistically differ from settlement on the nanoparticle-coated plugs (p = 0.014; Fig. 4; Supplementary Tables S9, S10 and S11).

#### Settlement: partially-coated plugs

**Figure 5:**
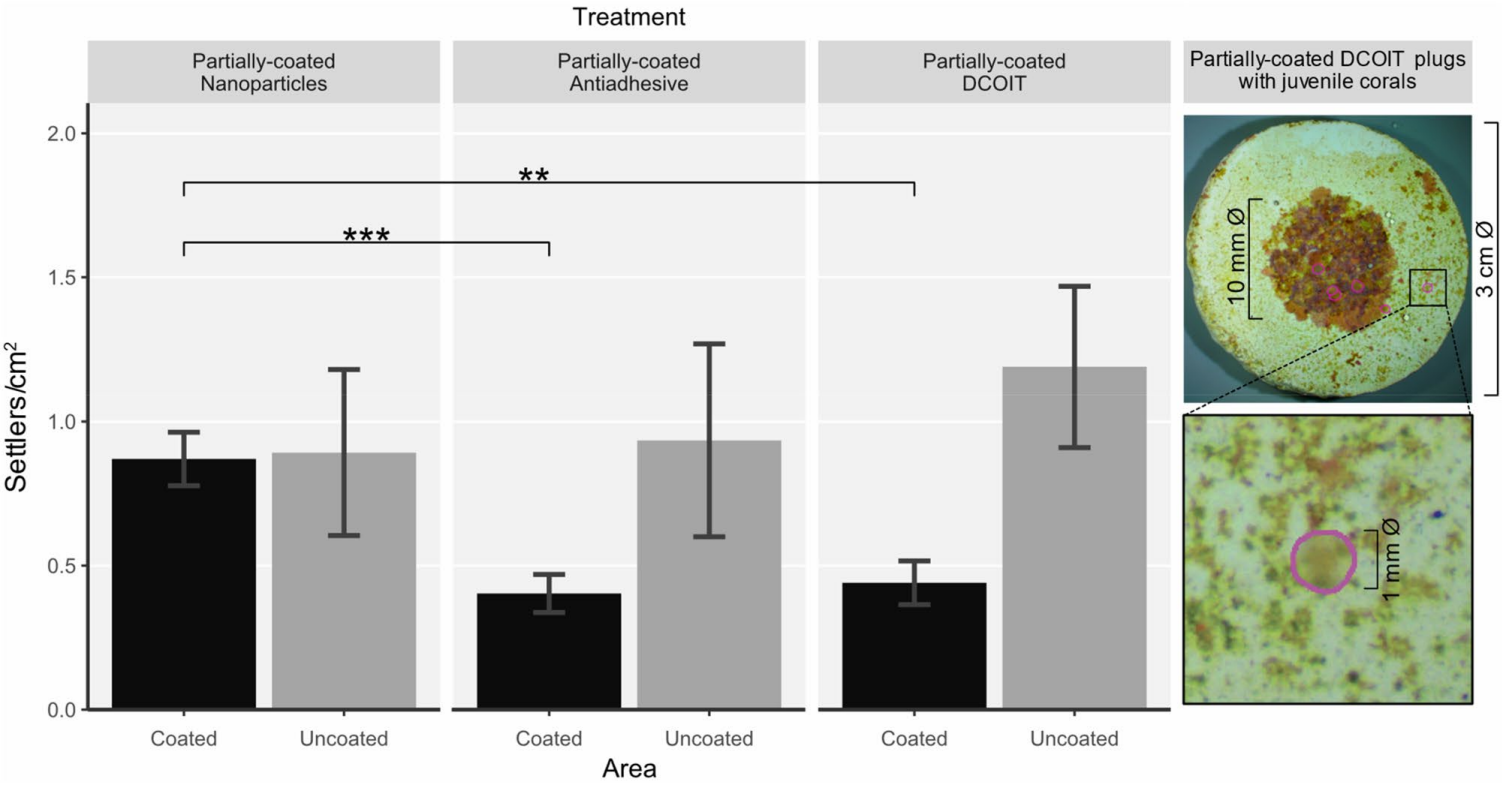
Pre-conditioned partially-coated (PC) plugs (Nanoparticles, Antiadhesive, DCOIT) with coated and uncoated areas (n = 30 plugs per treatment) were tested for mean coral larval settlement (settlers per cm^2^) in individual glass jars (one plug with 15 larvae per jar). Error bars represent SEM. Asterisks indicate statistically significant differences based on pairwise post-hoc tests with estimated marginal means (Supplementary Tables S12 and S14; *p < 0.05, **p < 0.01, ***p < 0.001). Images (right) show coral settlers (circled in magenta) on a PC DCOIT plug.

Mean settlement densities (settlers/cm^2^) differed significantly among treatments and between areas (coated/uncoated) (LMM: χ^2^(2) = 6.6274, p < 0.05), and settlement patterns on the PC plugs were similar to the FC plugs. Settlement on the coated NP areas, with 0.9 ± 0.1 settlers/cm^2^ (mean ± SE), was significantly higher than settlement on the coated antiadhesive areas (p < 0.001) and coated DCOIT areas (p = 0.004) (Fig. 5; Supplementary Tables S12 and S14). *A. tenuis* settlement on the uncoated areas of the antiadhesive coating (0.9 ± 0.3 m settlers/cm^2^) and DCOIT coating (1.2 ± 0.3 settlers/cm^2^) was much higher than on the coated areas in close vicinity on the same plugs, however, not significantly (Fig. 5; Supplementary Tables S12, S13 and S14).

## Discussion

The effects of non-biocidal and recently-developed antifouling (AF) coatings on marine species have been rarely evaluated since their introduction after 2008, when tributyltin (TBT) was globally prohibited^92^. By contrast, DCOIT, as a biocidal ingredient in a range of AF coatings, has been investigated and evaluated to a greater extent, but with no testing of hard corals or coral larvae to date. Here, three coatings with reportedly high AF efficacy and potential low toxicity^64,66,68^ were tested, to explore whether any of these coatings caused reduced algae growth without reducing coral larval settlement (indicating low deterrent or toxic effects). Two of the three AF coatings, the encapsulated DCOIT and antiadhesive coating, showed effective and localized antifouling action, while the CeO_2−x_ coating formulation was ineffective. Coral settlement on the fully-coated (FC) plugs of all treatments did not differ from settlement on the control; however, coral larvae tended to settle on an uncoated area if given the choice (in partially-coated (PC) antiadhesive and DCOIT treatments), leading to higher settler densities in that area. These results indicate that larval settlement on and around the AF coatings is possible, and that the AF coatings can reduce fouling and competition with newly settled corals.

### Antifouling efficacy and *A. tenuis* settlement: CeO_2−x_ nanoparticle coating

The CeO_2−x_ nanoparticle (NP) coating did not actively inhibit fouling or change fouling community composition compared with the uncoated control. This contrasted with Herget et al.^68^ and Wu et al.^93^ who demonstrated beneficial antifouling of stainless-steel plates coated with CeO_2−x_ NP in freshwater and seawater environments. The enzymatic activity of the CeO_2−x_ nanoparticles (NPs) catalyzes the oxidation of halides in presence of hydrogen peroxide (H_2_O_2_) to hypohalous acids (HOX) according to: Br^-^ + H_2_O_2_ → HOBr + H_2_O. HOX can react in follow-up reactions to halogenated organic compounds. Hypohalous acids combat biofilm formation through their biocidal activity or the formation of signaling molecules involved in intracellular communication. When cell-to-cell communication is blocked by halogenated compounds, bacteria cannot form organized community structures such as biofilms. As the catalytic activity of the NPs was tested in their unconsolidated form, we assumed their activity in the coating would still be present. Future studies could test this activity in the coating just prior to any field tests. Furthermore, the concentration of NPs in our coating could have been too low for the expected antifouling activity and future approaches could test for different NP concentrations in the coatings. Although several studies report substantial reductions in bacterial adhesion^68,93–95^, literature on suppressed macrofouling species in natural seawater is very scarce^96^. Interestingly, Xu et al.^97^ reported increased biofilm formation and growth of bacterial strains from wastewater treatment plants at CeO_2−x_ NP concentrations < 4 mg/L, whereas the opposite effect was observed at CeO_2−x_ NP concentrations higher than 10 mg/L. Concentrations of 0.5 and 2 mg/L were reported to enhance the surface hydrophobicity, aggregating ability, and the α-, and β-d-glucopyranose polysaccharide production of the bacterial strains. In addition, Xu et al.^97,98^ reported similar findings of ROS-induced acceleration of quorum sensing molecules and activation of inherent resistance capacity by bacteria. Thus, ROS generated by non-lethal CeO_2−x_ NP concentrations may accelerate bacterial biofouling and subsequent macrofouling. Clearly more studies to optimise the composition of NP antifoulants are needed to assess their potential to reduce fouling in seawater under tropical conditions.

In our study, *A. tenuis* larval settlement on the CeO_2−x_ NP coated surfaces was higher than on the uncoated control, and was significantly higher on the FC plugs and coated PC plugs than on the antiadhesive- or DCOIT-coating. Also, when given the choice on PC plugs, larvae chose to settle on both coated and uncoated areas equally. However, ROS-mediated manipulation by the CeO_2−x_ NPs could have created subtle effects, or effects that cancelled each other out in terms of settlement outcomes. For example, there may have been positive or negative effects of generated ROS on both the fouling community beyond the resolution of this study and there could have been effects directly on the larvae as they contacted the surface in their search for a site to settle.

### Antifouling efficacy and *A. tenuis* settlement: Antiadhesive coating

The antiadhesive coating showed the second highest AF efficacy, although it was much lower than that of the DCOIT coating. Our findings are in agreement with previous reports on the same or similar sol–gel coating types. For example, Tang et al.^99^ found inhibition of zoospore settlement of the marine fouling alga *Ulva linza* after exposure to turbulent flow on sol–gel-derived xerogel films with *n*-octyltriethoxysilane (C8), tetraethylorthosilane (TEOS) and tetramethylorthosilane (TMOS), similar to the constituents of the coatings used in the present study. Also, their xerogel was found to act as a fouling-release surface for juveniles of the tropical barnacle *Balanus amphitrite*. Similarly, Gunari et al.^100^ showed good AF results with *n*-octadecyl-trimethoxysilane (C18), C8 and TEOS on the settlement of barnacle cyprids, removal of juvenile barnacles (*B. amphitrite*) and settlement of zoospores of the alga *U. linza*. Sokolova et al.^74^ tested an identical sol–gel xerogel coating (1:4:45:50; C18/TDF/C8/TEOS) containing the same constituents in the same molar ratios as tested here. That study found higher releases of juvenile barnacles and *Ulva* sp. sporelings, as compared with the C18/C8/TEOS formulation^100^. The C18/TDF/C8/TEOS xerogel contributes to the increased fraction of barnacles removed via shear^66^.

However, Sokolova et al.^74^ pointed out that sometimes opposing patterns in the settlement and release of different fouling organisms make it difficult to design a single surface to minimize settlement of all fouling organisms^99^. The goal of our study was to identify an AF coating that potentially inhibited harmful (i.e. competitive) fouling, while not deterring coral larval settlement (i.e. not inhibiting the sensitive settlement process). The antiadhesive sol–gel performed very well to inhibit CCA and green/brown algae but was also one of the two coatings that significantly reduced coral settlement, as compared to the highest settlement found on the nanoparticle treatment. In the choice experiment, *A. tenuis* settlement was approximately 50% lower on the coated areas of the PC antiadhesive plugs than on the uncoated areas (although not statistically significant). This could indicate disadvantageous surface properties of the coating for coral larval settlement. Unlike the DCOIT coating, sol–gel does not contain a known biocide, so the inhibition of settlement is likely to be physical, and any unlikely potential for deterrent or toxic effects should be investigated by assessing the survival of spat on, and adjacent to, the sol–gel coating. Interestingly, the abundance of CCA on the antiadhesive sol–gel coating was no different from the control, suggesting that the sol–gel coating might create a favorable environment for CCA growth and could be further investigated as a method to encourage the colonization of surfaces with CCA that are advantageous for larval settlement in coral propagation and supply for restoration programs.

### Antifouling efficacy and *A. tenuis* settlement: encapsulated DCOIT-coating

To date, very few studies have assessed the effects of encapsulated DCOIT in tropical marine species^65^. Even less is known about the effects of encapsulated DCOIT integrated in a coating, as compared to its free form, on marine species^64^. The rationale behind the encapsulation/immobilization technology is the prevention of direct biocidal interaction with coating components, the control of leaching rate, and thus, the decrease of the absolute quantity of biocides needed to prepare a formulation with identical AF efficacy, increasing the coating lifetime and reducing potential environmental threats^64,101–103^. Only one study by Ferreira et al.^62^ compared the effects of free (non-integrated in a coating) and encapsulated DCOIT on the soft coral *Sarcophyton glaucum*. The authors found reduced toxicity of encapsulated DCOIT on the coral fragments compared with free DCOIT, as measured by coral polyp retraction, photosynthetic efficacy, and oxidative-stress markers. Our study demonstrated the high AF efficacy of encapsulated DCOIT with a very localized area of effect. The biofouling analysis showed clear differences in fouling between coated and uncoated areas on the same plugs, indicating that antifouling activity was isolated to the coating’s surface with negligible leaching of antifoulants onto adjacent uncoated areas (1–3 mm away).

In this study, the settlement of *A. tenuis* directly on the FC DCOIT-coated areas was not significantly lower than settlement on the control plugs. These results suggest that either the coating has generally low deterrent or toxic effects, or that the potential toxicity declined sufficiently during the 37 days of pre-conditioning so as not to impede settlement. Inhibition of larval settlement is very sensitive to contaminants, including the AF biocides copper and TBT^50,104^, and the lack of response to DCOIT in this no-choice experiment is strong evidence that the coating was not harmful at the time of the assay. Nevertheless, when larvae were able to choose between DCOIT-coated and uncoated surfaces (on PC plugs), almost three times more larvae chose to settle on the uncoated surface of PC plugs (per cm^2^). It is possible that this was due to a subtle influence of the DCOIT itself (e.g., toxicity or textural), but these results could be further investigated by focusing on freshly applied DCOIT, as well as leaching rates and spatial and temporal effects on the physiological, and molecular responses of the larvae. Alternatively, the uncoated area of PC plugs may have developed a stronger inductive microbial biofilm for larval settlement^18^ independently of, or influenced by, the adjacent DCOIT coating. Future research could investigate fouling organisms quantitatively, including bacterial species abundances and specific taxonomic occurrences on and next to the coatings. Moreover, long-term survival of coral spat and the associated benthic community on and next to the coating could be investigated.

The DCOIT coating worked particularly well to suppress CCA growth. With 1% coverage on the FC plugs and 5% on the coated areas of the PC plugs, CCA growth was strongly inhibited as compared to the control plugs with 31% CCA, and the uncoated areas with 23% CCA (PC plugs) after 37 days. This finding could indicate high toxicity towards CCA and could be particularly relevant for industrial maritime sectors. Although many studies have shown the importance of CCA as a cue for coral settlement^19,23,105^, studies have demonstrated that CCA can also outcompete coral spat and lead to spat mortality under certain conditions^106,107^. Therefore, control of CCA overgrowth is an important consideration for improving survival in coral propagation for restoration purposes^38^. Our results support those of Figueiredo et al.^108^ who demonstrated good AF performance of encapsulated DCOIT with reduced toxicity towards non-target species, including microalgae, rotifers, bivalves, crustaceans and echinoderms.

## Conclusion and future considerations

Two of the three types of AF coatings successfully inhibited macroalgal competitors of coral spat in mesocosm aquaria over 37 days. Our results also suggest no detrimental effects of the 37-day old coatings on coral larvae during settlement. However, these findings could be further evaluated by testing freshly applied coatings and determining possible toxic leaching effects in time-series experiments lasting for several weeks to months. Now that promising AF candidates have been identified, further studies could be conducted to assess their potential benefits on the survival and growth of coral spat over the first months post-settlement. Tebben et al.^48^ demonstrated that a non-toxic paraffin wax-based AF was able to improve the survival of early coral spat that were in close proximity (within mm) of the wax coating. A similar approach of settling larvae directly onto a deployment substrate immediately adjacent to, or surrounded by, DCOIT or antiadhesive AF coatings, may offer strong protection against overgrowth by algae. While biocide-containing AFs (Cu_2_O) have been effective in reducing fouling in coral fragments in nurseries for reef restoration^109^, the biocide-free antiadhesive is likely to be more suitable for application on coral settlement surfaces for deployment into the field where biocides such as Cu_2_O or DCOIT are prohibited.

## Supplementary Information

Supplementary Information.

## Data Availability

The raw data of the fouling quantification with the Trainable Weka Segmentation in ImageJ can be accessed publicly on-line here: https://doi.org/10.25845/ws1n-ah16.

## References

[1] Gardner TA, Côté IM, Gill JA, Grant A, Watkinson AR (2003). Long-term region-wide declines in Caribbean corals. Science.

[2] Bruno JF, Selig ER (2007). Regional decline of coral cover in the Indo-Pacific: Timing, extent, and subregional comparisons. PLoS ONE.

[3] De’Ath G, Fabricius KE, Sweatman H, Puotinen M (2012). The 27-year decline of coral cover on the Great Barrier Reef and its causes. Proc. Natl. Acad. Sci. U. S. A..

[4] Hughes TP, Graham NAJ, Jackson JBC, Mumby PJ, Steneck RS (2010). Rising to the challenge of sustaining coral reef resilience. Trends Ecol. Evol..

[5] Pandolfi JM, Connolly SR, Marshall DJ, Cohen AL (2011). Projecting coral reef futures under global warming and ocean acidification. Science.

[6] Hughes TP (2017). Global warming and recurrent mass bleaching of corals. Nature.

[7] Bindoff, N. L. *et al. Chapter 5: Changing Ocean, Marine Ecosystems, and Dependent Communities*. *IPCC Special Report on the Ocean and Cryosphere in a Changing Climate* (2019).

[8] Richmond RH, Birkeland CE (1997). Reproduction and recruitment in corals: Critical links in the persistence of reefs. Life and Death of Coral Reefs.

[9] Trapon ML, Pratchett MS, Hoey AS, Baird AH (2013). Influence of fish grazing and sedimentation on the early post-settlement survival of the tabular coral *Acropora cytherea*. Coral Reefs.

[10] Gallagher C, Doropoulos C (2017). Spatial refugia mediate juvenile coral survival during coral–predator interactions. Coral Reefs.

[11] Vermeij MJA, Sandin SA (2008). Density-dependent settlement and mortality structure the earliest life phases of a coral population. Ecology.

[12] Vermeij MJA, Smith JE, Smith CM, Vega Thurber R, Sandin SA (2009). Survival and settlement success of coral planulae: Independent and synergistic effects of macroalgae and microbes. Oecologia.

[13] Ricardo GF, Jones RJ, Nordborg M, Negri AP (2017). Settlement patterns of the coral *Acropora millepora* on sediment-laden surfaces. Sci. Total Environ..

[14] Brunner CA, Uthicke S, Ricardo GF, Hoogenboom MO, Negri AP (2021). Climate change doubles sedimentation-induced coral recruit mortality. Sci. Total Environ..

[15] Birrell CL, McCook LJ, Willis BL, Diaz-Pulido GA (2008). Effects of benthic algae on the replenishment of corals and the implications for the resilience of coral reefs. Oceanography and Marine Biology: An Annual Review.

[16] Karcher DB (2020). Nitrogen eutrophication particularly promotes turf algae in coral reefs of the central Red Sea. PeerJ.

[17] Kirschner CM, Brennan AB (2012). Bio-inspired antifouling strategies. Annu. Rev. Mater. Res..

[18] Webster NS (2004). Metamorphosis of a scleractinian coral in response to microbial biofilms. Appl. Environ. Microbiol..

[19] Heyward AJ, Negri AP (1999). Natural inducers for coral larval metamorphosis. Coral Reefs.

[20] Negri AP, Webster NS, Hill RT, Heyward AJ (2001). Metamorphosis of broadcast spawning corals in response to bacteria isolated from crustose algae. Mar. Ecol. Prog. Ser..

[21] Tebben J (2011). Induction of larval metamorphosis of the coral *Acropora millepora* by tetrabromopyrrole isolated from a *Pseudoalteromonas bacterium*. PLoS ONE.

[22] Sneed JM, Sharp KH, Ritchie KB, Paul VJ (2014). The chemical cue tetrabromopyrrole from a biofilm bacterium induces settlement of multiple Caribbean corals. Proc. R. Soc. B Biol. Sci..

[23] Tebben J (2015). Chemical mediation of coral larval settlement by crustose coralline algae. Sci. Rep..

[24] Carpenter RC, Edmunds PJ (2006). Local and regional scale recovery of *Diadema* promotes recruitment of scleractinian corals. Ecol. Lett..

[25] Box SJ, Mumby PJ (2007). Effect of macroalgal competition on growth and survival of juvenile Caribbean corals. Mar. Ecol. Prog. Ser..

[26] Linares C, Cebrian E, Coma R (2012). Effects of turf algae on recruitment and juvenile survival of gorgonian corals. Mar. Ecol. Prog. Ser..

[27] McCook LJ, Jompa J, Diaz-Pulido G (2001). Competition between corals and algae on coral reefs: A review of evidence and mechanisms. Coral Reefs.

[28] Nugues MM, Smith GW, Van Hooidonk RJ, Seabra MI, Bak RPM (2004). Algal contact as a trigger for coral disease. Ecol. Lett..

[29] Fong J (2019). Allelopathic effects of macroalgae on Pocillopora acuta coral larvae. Mar. Environ. Res..

[30] Hauri C, Fabricius KE, Schaffelke B, Humphrey C (2010). Chemical and physical environmental conditions underneath mat- and canopy-forming macroalgae, and their effects on understorey corals. PLoS ONE.

[31] Bay, L. K. *et al. Reef Restoration and Adaptation Program : Intervention Technical Summary. A report provided to the Australian Government by the Reef Restoration and Adaptation Program*. (2019).

[32] Anthony KRN (2020). Interventions to help coral reefs under global change—A complex decision challenge. PLoS ONE.

[33] Vardi T (2021). Six priorities to advance the science and practice of coral reef restoration worldwide. Restor. Ecol..

[34] Heyward AJ, Rees M, Smith LD (1999). Coral spawning slicks harnessed for large-scale coral culture. Progr. Abstr. Int. Conf. Sci. Asp. Coral Reef Assess. Monit. Restor..

[35] Harrison P, Villanueva R, De la Cruz D (2016). Coral Reef Restoration using Mass Coral Larval Reseeding.

[36] de la Cruz DW, Harrison PL (2020). Enhancing coral recruitment through assisted mass settlement of cultured coral larvae. PLoS ONE.

[37] Chamberland VF, Snowden S, Marhaver KL, Petersen D, Vermeij MJA (2017). The reproductive biology and early life ecology of a common Caribbean brain coral, *Diploria labyrinthiformis* (Scleractinia: Faviinae). Coral Reefs.

[38] Randall CJ (2020). Sexual production of corals for reef restoration in the Anthropocene. Mar. Ecol. Prog. Ser..

[39] Miller MW (2021). Settlement yields in large-scale in situ culture of Caribbean coral larvae for restoration. Restor. Ecol..

[40] Baria-Rodriguez MV, de la Cruz DW, Dizon RM, Yap HT, Villanueva RD (2019). Performance and cost-effectiveness of sexually produced *Acropora granulosa* juveniles compared with asexually generated coral fragments in restoring degraded reef areas. Aquat. Conserv. Mar. Freshw. Ecosyst..

[41] Doropoulos C, Elzinga J, ter Hofstede R, van Koningsveld M, Babcock RC (2019). Optimizing industrial-scale coral reef restoration: Comparing harvesting wild coral spawn slicks and transplanting gravid adult colonies. Restor. Ecol..

[42] Kuffner IB, Andersson AJ, Jokiel PL, Rodgers KS, MacKenzie FT (2008). Decreased abundance of crustose coralline algae due to ocean acidification. Nat. Geosci..

[43] Webster NS, Uthicke S, Botté ES, Flores F, Negri AP (2013). Ocean acidification reduces induction of coral settlement by crustose coralline algae. Glob. Change Biol..

[44] Randall CJ, Giuliano C, Heyward AJ, Negri AP (2021). Enhancing coral survival on deployment devices with microrefugia. Front. Mar. Sci..

[45] Kuffner IB (2006). Inhibition of coral recruitment by macroalgae and cyanobacteria. Mar. Ecol. Prog. Ser..

[46] Arnold SN, Steneck RS, Mumby PJ (2010). Running the gauntlet: Inhibitory effects of algal turfs on the processes of coral recruitment. Mar. Ecol. Prog. Ser..

[47] Speare KE, Duran A, Miller MW, Burkepile DE (2019). Sediment associated with algal turfs inhibits the settlement of two endangered coral species. Mar. Pollut. Bull..

[48] Tebben J, Guest JR, Sin TM, Steinberg PD, Harder T (2014). Corals like it waxed: Paraffin-based antifouling technology enhances coral spat survival. PLoS ONE.

[49] Almeida E, Diamantino TC, de Sousa O (2007). Marine paints: The particular case of antifouling paints. Prog. Org. Coat..

[50] Negri AP, Smith LD, Webster NS, Heyward AJ (2002). Understanding ship-grounding impacts on a coral reef: Potential effects of anti-foulant paint contamination on coral recruitment. Mar. Pollut. Bull..

[51] Smith LD, Negri AP, Philipp E, Webster NS, Heyward AJ (2003). The effects of antifoulant-paint-contaminated sediments on coral recruits and branchlets. Mar. Biol..

[52] Jacobson AH, Willingham GL (2000). Sea-nine antifoulant: An environmentally acceptable alternative to organotin antifoulants. Sci. Total Environ..

[53] Silva V (2020). Isothiazolinone biocides: Chemistry, biological, and toxicity profiles. Molecules.

[54] da Silva AR, da Guerreiro AS, Martins SE, Sandrini JZ (2021). DCOIT unbalances the antioxidant defense system in juvenile and adults of the marine bivalve Amarilladesma mactroides (Mollusca: Bivalvia). Comp. Biochem. Physiol. Part C.

[55] Cima F (2013). Preliminary evaluation of the toxic effects of the antifouling biocide Sea-Nine 211^TM^ in the soft coral *Sarcophyton cf. glaucum* (Octocorallia, Alcyonacea) based on PAM fluorometry and biomarkers. Mar. Environ. Res..

[56] Wendt I, Backhaus T, Blanck H, Arrhenius Å (2016). The toxicity of the three antifouling biocides DCOIT, TPBP and medetomidine to the marine pelagic copepod *Acartia tonsa*. Ecotoxicology.

[57] Chen L (2017). Identification of molecular targets for 4,5-dichloro-2-n-octyl-4-isothiazolin-3-one (DCOIT) in teleosts: New insight into mechanism of toxicity. Environ. Sci. Technol..

[58] Martins SE, Fillmann G, Lillicrap A, Thomas KV (2018). Review: Ecotoxicity of organic and organo-metallic antifouling co-biocides and implications for environmental hazard and risk assessments in aquatic ecosystems. Biofouling.

[59] Moon YS, Kim M, Hong CP, Kang JH, Jung JH (2019). Overlapping and unique toxic effects of three alternative antifouling biocides (Diuron, Irgarol 1051 ®, Sea-Nine 211 ® ) on non-target marine fish. Ecotoxicol. Environ. Saf..

[60] Su Y (2019). Toxicity of 4,5-dichloro-2-n-octyl-4-isothiazolin-3-one (DCOIT) in the marine decapod *Litopenaeus vannamei*. Environ. Pollut..

[61] Fonseca VB, da Guerreiro AS, Vargas MA, Sandrini JZ (2020). Effects of DCOIT (4,5-dichloro-2-octyl-4-isothiazolin-3-one) to the haemocytes of mussels *Perna perna*. Comp. Biochem. Physiol Part C.

[62] Ferreira V (2021). Effects of nanostructure antifouling biocides towards a coral species in the context of global changes. Sci. Total Environ..

[63] de Campos BG (2022). A preliminary study on multi-level biomarkers response of the tropical oyster *Crassostrea brasiliana* to exposure to the antifouling biocide DCOIT. Mar. Pollut. Bull..

[64] Maia F (2015). Incorporation of biocides in nanocapsules for protective coatings used in maritime applications. Chem. Eng. J..

[65] Santos JVN (2020). Can encapsulation of the biocide DCOIT affect the anti-fouling efficacy and toxicity on tropical bivalves?. Appl. Sci..

[66] Detty MR, Ciriminna R, Bright FV, Pagliaro M (2014). Environmentally benign sol-gel antifouling and foul-releasing coatings. Acc. Chem. Res..

[67] Korschelt K, Tahir MN, Tremel W (2018). A Step into the future: Applications of nanoparticle enzyme mimics. Chemistry.

[68] Herget K (2017). Haloperoxidase mimicry by CeO2-x nanorods combats biofouling. Adv. Mater..

[69] Korschelt K (2018). CeO2-: X nanorods with intrinsic urease-like activity. Nanoscale.

[70] Herget K, Frerichs H, Pfitzner F, Tahir MN, Tremel W (2018). Functional enzyme mimics for oxidative halogenation reactions that combat biofilm formation. Adv. Mater..

[71] Doropoulos C, Ward S, Marshell A, Diaz-Pulido G, Mumby PJ (2012). Interactions among chronic and acute impacts on coral recruits: The importance of size-escape thresholds. Ecology.

[72] Ji Z (2012). Designed synthesis of CeO2 nanorods and nanowires for studying toxicological effects of high aspect ratio nanomaterials. ACS Nano.

[73] Herget K (2017). Supporting Information: Haloperoxidase mimicry by CeO2-x nanorods combats biofouling. Adv. Mater..

[74] Sokolova A (2012). Spontaneous multiscale phase separation within fluorinated xerogel coatings for fouling-release surfaces. Biofouling.

[75] Schneider CA, Rasband WS, Eliceiri KW (2012). NIH Image to ImageJ: 25 years of image analysis. Nat. Methods.

[76] *ImageJ Release Notes*. https://imagej.nih.gov/ij/notes.html.

[77] Arganda-Carreras I (2017). Trainable Weka Segmentation: A machine learning tool for microscopy pixel classification. Bioinformatics.

[78] Arganda-Carreras, I. *et al.**Supplementary Data: Trainable Weka Segmentation: A Machine Learning Tool for Microscopy Pixel Classification: Trainable Weka Segmentation User Manual*10.1093/bioinformatics/btx180 (2017). 10.1093/bioinformatics/btx180 28369169

[79] Vyas N, Sammons RL, Addison O, Dehghani H, Walmsley AD (2016). A quantitative method to measure biofilm removal efficiency from complex biomaterial surfaces using SEM and image analysis. Sci. Rep..

[80] Carbone DA, Gargano I, Pinto G, De Natale A, Pollio A (2017). Evaluating microalgae attachment to surfaces: A first approach towards a laboratory integrated assessment. Chem. Eng. Trans..

[81] Moreno Osorio JH (2020). Early colonization stages of fabric carriers by two Chlorella strains. J. Appl. Phycol..

[82] Ricardo GF (2021). Impacts of water quality on *Acropora* coral settlement: The relative importance of substrate quality and light. Sci. Total Environ..

[83] Macadam A, Nowell CJ, Quigley K (2021). Machine learning for the fast and accurate assessment of fitness in coral early life history. Remote Sens..

[84] Negri AP, Heyward AJ (2000). Inhibition of Fertilization and Larval Metamorphosis of the Coral *Acropora millepora* (Ehrenberg, 1834) by Petroleum Products. Mar. Pollut. Bull..

[85] Nordborg FM, Flores F, Brinkman DL, Agustí S, Negri AP (2018). Phototoxic effects of two common marine fuels on the settlement success of the coral *Acropora tenuis*. Sci. Rep..

[86] R Core Team. *R: A Language and Environment for Statistical Computing*. (2021).

[87] Wickham H (2019). Welcome to the Tidyverse. J. Open Source Softw..

[88] Pinheiro, J., Bates, D., Debroy, S., Sarkar, D. & R Core Team (2021). Linear and nonlinear mixed effects models contact. Linear nonlinear Mix. Eff. Model..

[89] Fox J, Weisberg S (2019). An R Companion to Applied Regression.

[90] Lenth, R. V. *Emmeans: Estimated Marginal Means*. https://cran.r-project.org/package=emmeans (2021).

[91] Brooks ME (2017). glmmTMB balances speed and flexibility among packages for zero-inflated generalized linear mixed modeling. R J..

[92] Dafforn KA, Lewis JA, Johnston EL (2011). Antifouling strategies: History and regulation, ecological impacts and mitigation. Mar. Pollut. Bull..

[93] Wu R (2021). Room temperature synthesis of defective cerium oxide for efficient marine anti-biofouling. Adv. Compos. Hybrid Mater..

[94] Hu M (2018). Nanozymes in nanofibrous mats with haloperoxidase-like activity to combat biofouling. ACS Appl. Mater. Interfaces.

[95] He X (2020). Haloperoxidase mimicry by CeO2-x nanorods of different aspect ratios for antibacterial performance. ACS Sustain. Chem. Eng..

[96] Saxena P, Harish (2018). Nanoecotoxicological reports of engineered metal oxide nanoparticles on algae. Curr. Pollut. Rep..

[97] Xu Y (2019). Effects of cerium oxide nanoparticles on bacterial growth and behaviors: Induction of biofilm formation and stress response. Environ. Sci. Pollut. Res..

[98] Xu Y (2018). Mechanistic understanding of cerium oxide nanoparticle-mediated biofilm formation in Pseudomonas aeruginosa. Environ. Sci. Pollut. Res..

[99] Tang Y (2005). Hybrid xerogel films as novel coatings for antifouling and fouling release. Biofouling.

[100] Gunari N (2011). The control of marine biofouling on xerogel surfaces with nanometer-scale topography. Biofouling.

[101] Maia F (2012). Silica nanocontainers for active corrosion protection. Nanoscale.

[102] Martins R (2017). Effects of a novel anticorrosion engineered nanomaterial on the bivalve: *Ruditapes philippinarum*. Environ. Sci. Nano.

[103] Gutner-Hoch E (2018). Antimacrofouling efficacy of innovative inorganic nanomaterials loaded with booster biocides. J. Mar. Sci. Eng..

[104] Negri AP, Heyward AJ (2001). Inhibition of coral fertilisation and larval metamorphosis by tributyltin and copper. Mar. Environ. Res..

[105] Morse DE, Hooker N, Morse ANC, Jensen RA (1988). Control of larval metamorphosis and recruitment in sympatric agariciid corals. J. Exp. Mar. Biol. Ecol..

[106] Harrington L, Fabricius K, De’ath G, Negri A (2004). Recognition and selection of settlement substrata determine post-settlement survival in corals. Ecology.

[107] Jorissen H, Baumgartner C, Steneck RS, Nugues MM (2020). Contrasting effects of crustose coralline algae from exposed and subcryptic habitats on coral recruits. Coral Reefs.

[108] Figueiredo J (2019). Toxicity of innovative anti-fouling nano-based solutions to marine species. Environ. Sci. Nano.

[109] Shafir S, Abady S, Rinkevich B (2009). Improved sustainable maintenance for mid-water coral nursery by the application of an anti-fouling agent. J. Exp. Mar. Biol. Ecol..

