# Supplementary material for: Antifouling coatings can reduce algal growth while preserving coral settlement
Source: Sci Rep. 2022 Sep 24;12:15935. doi: 10.1038/s41598-022-19997-6 (PMC9509345; doi:10.1038/s41598-022-19997-6)
Supplement: Supplementary file 1 — Supplementary Information. [file 41598_2022_19997_MOESM1_ESM.docx]

**Supplementary Information: Antifouling coatings can reduce algal growth while preserving coral settlement**

Lisa K. Roepke ^1*^, David Brefeld ^2^, Ulrich Soltmann ^3^, Carly J. Randall ^4^, Andrew P. Negri ^4^, Andreas Kunzmann ^1^

^1^ Leibniz Centre for Tropical Marine Research, Bremen, Germany

^2^ Institut für Chemie und Biologie des Meeres, Carl-von-Ossietzky Universität Oldenburg, Wilhelmshaven, Germany

^3^ Gesellschaft zur Förderung von Medizin-, Bio- und Umwelttechnologien e.V., Dresden, Germany

^4^ Australian Institute of Marine Science, PMB 3, Townsville, Queensland 4810, Australia

*Correspondence to: lisa.roepke@leibniz-zmt.de

**SI Materials and Methods**

CeO_2-x_ nanoparticle coating

(i) The production of nanoparticles

An aqueous solution of NaOH (100 mL, 9.0 M, 37.8 g) was directly poured into a rapidly stirred solution of cerium(III) nitrate hexahydrate (20 mL, 5.2 g, 0.05 M) in MilliQ-water. After being vigorously stirred for 30 min, the lilac suspension was transferred into a stainless steel autoclave with Teflon insert (total volume: 300 mL) and heated to 100 °C for 24 hours. The lilac product was separated from the turbid suspension by centrifugation (3000 rpm, 10 min) and washed two times with MilliQ-water (15 mL each). After drying at 60 °C overnight, the resulting yellow product was ground in a vibrating ball mill for two minutes at 30 rpm. The milling yielded agglomerates of the size < 20 µm.

(ii) The catalytic activity testing

For catalytic activity testing, 25 µg of NPs in 1 mL MilliQ-water were mixed with a vortex oscillator for 3 sec and exposed to ultrasound (Bandelin electronic GmbH & Co. KG, SONOPULS HD 2070.2 Homogenisator with MS 72) twice (15 sec each). The catalytic haloperoxidase-like activity of the NPs was successfully demonstrated using the phenol red (PR; Sigma Aldrich; CAS 143-74-8) bromination assay following the methods and optimized parameters in Table S3 in Herget *et al.* (2017) ^(1,2)^. Spectrophotometrically measured samples containing ﻿CeO_2−x_ NPs^a^, PR^b^, NH_4_Br (Sigma Aldrich; CAS 12124-97-9)^c^ and ﻿H_2_O_2_ (Sigma Aldrich; CAS 7722-84-1)^d^ were added up to a volume of 300 µl (7 µl ^a^, 47 µl ^b^, 186 µl ^c^, 60 µl ^d^).

(iii) The production of coating solution A

Prior to coating, the plugs were cleaned with ethanol and activated using microwave plasma (as above). Two different coating solutions were produced. Coating solution A was made from 100 mL of tetraethoxysilane (TEOS) and mixed with 420 mL of ethanol (96%) and 20 mL of 0.01 M HCl and stirred overnight at room temperature for hydrolysis. An acidic silica nano-sol with a solids content of about 6 wt% was formed.

(iiii) the production of coating solution B

Coating solution B was made from 75 g tetraethoxysilane (TEOS), 25 g 3-glycidyloxypropyltriethoxysilane (GLYEO), 420 mL ethanol (96%) and 20 mL 0.01 M HCl and stirred overnight at room temperature for hydrolysis. The solids content of coating solution B was also about 6 wt%.

30 mL (= 1.8 g SiO_2_) of each coating solution was mixed with 1 g of ceria (cerium dioxide) powder. Before adding the ceria NPs to the sols, the ceria powder was dispersed in MilliQ-water and the pH was adjusted to 7.5 with 1N HCl. A pH of about 5 was reached at which the ready-to-coat sols had a sufficient pot life of 1-2 days.

**SI Figures**

**
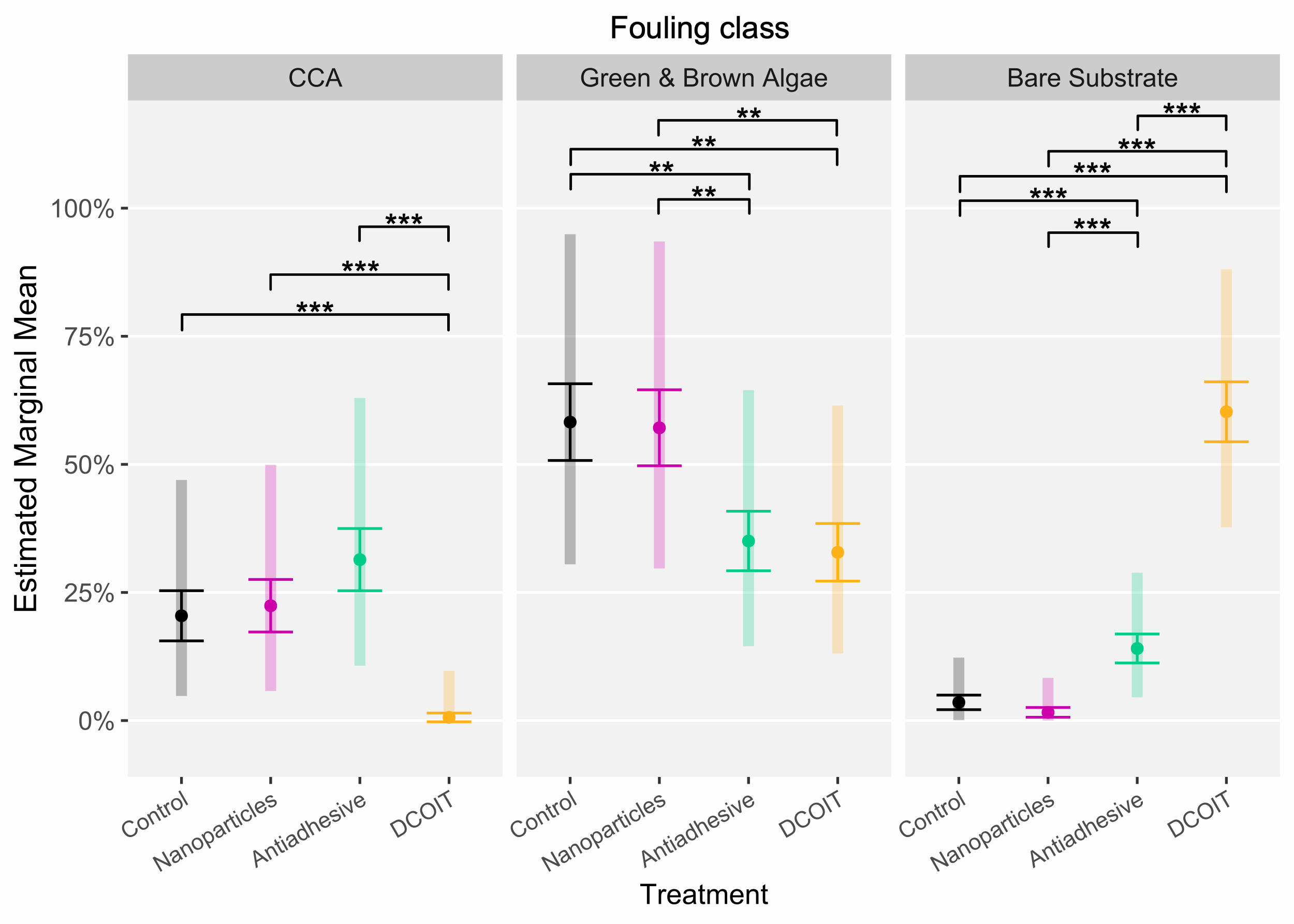
**

**Figure S1.** Estimated marginal means, SE and upper and lower confidence levels (CL) of the fouling classes on the fully-coated (FC) plugs after 37 days. Estimates were back-transformed from the square-root scale. Asterisks indicate statistically significant differences of pairwise post-hoc tests based on estimated marginal means (Supplementary Tables S2 and S3; *p < 0.05, **p < 0.01, ***p < 0.001).

**
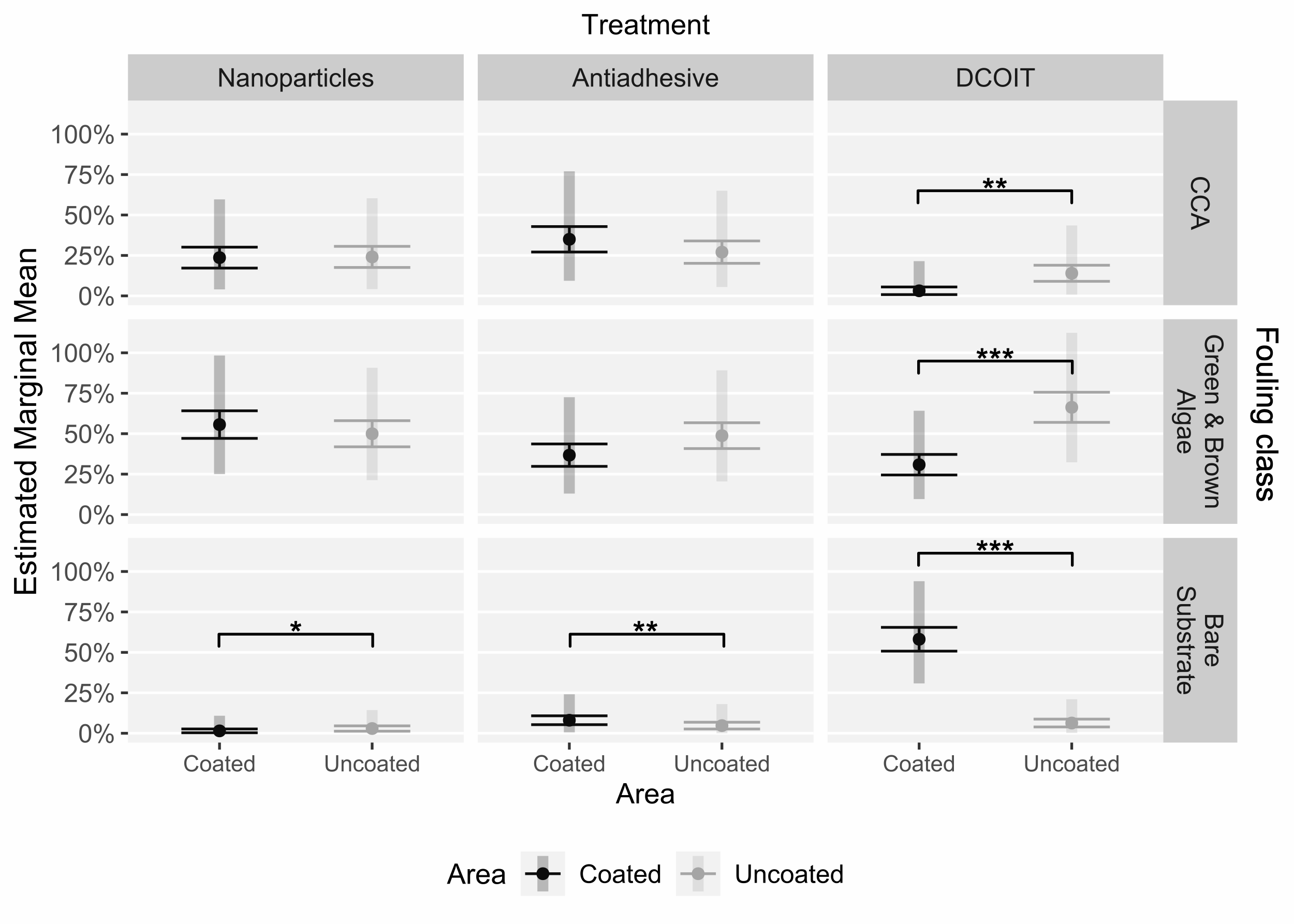
**

**Figure S2.** Estimated marginal means, SE and confidence levels of the fouling classes per area (coated/uncoated) on the partially-coated (PC) plugs after 37 days. Estimated were back-transformed from the square-root scale. Asterisks indicate statistically significant differences of pairwise post-hoc tests based on estimated marginal means (Supplementary Tables S5 and S7; *p < 0.05, **p < 0.01, ***p < 0.001).

**SI Tables**

**Table S1: Summary statistics of fouling coverage data for each fouling class (CCA, green/brown algae, bare substrate) and treatment (Control, Nanoparticles, Antiadhesive, DCOIT) combination at each monitoring period (9, 23, 37 days) on the fully-coated (FC) plugs.** Values represent mean percent fouling coverage (%). N (= 45) corresponds to the number of plugs in each treatment measured repeatedly for each monitoring period.

| **Fouling class** | **Days** | **Treatment** | **Mean fouling coverage** | **Standard error** |
| --- | --- | --- | --- | --- |
| CCA | 9 | Control | 0.5 | 0.1 |
| CCA | 23 | Control | 26.5 | 4.2 |
| CCA | 37 | Control | 30.7 | 5.2 |
| Green/brown Algae | 9 | Control | 50.4 | 5.1 |
| Green/brown Algae | 23 | Control | 66 | 4.8 |
| Green/brown Algae | 37 | Control | 64.8 | 5.1 |
| Bare Substrate | 9 | Control | 49.1 | 5.1 |
| Bare Substrate | 23 | Control | 7.5 | 1.1 |
| Bare Substrate | 37 | Control | 4.5 | 0.6 |
| CCA | 9 | Nanoparticles | 0.3 | 0 |
| CCA | 23 | Nanoparticles | 25.3 | 4.3 |
| CCA | 37 | Nanoparticles | 32.8 | 5.4 |
| Green/brown Algae | 9 | Nanoparticles | 54.7 | 5.4 |
| Green/brown Algae | 23 | Nanoparticles | 67 | 5 |
| Green/brown Algae | 37 | Nanoparticles | 65 | 5.3 |
| Bare Substrate | 9 | Nanoparticles | 45.1 | 5.5 |
| Bare Substrate | 23 | Nanoparticles | 7.7 | 1.6 |
| Bare Substrate | 37 | Nanoparticles | 2.3 | 0.4 |
| CCA | 9 | Antiadhesive | 0.4 | 0.1 |
| CCA | 23 | Antiadhesive | 22.1 | 3.3 |
| CCA | 37 | Antiadhesive | 40.3 | 5.3 |
| Green/brown Algae | 9 | Antiadhesive | 27.8 | 4.9 |
| Green/brown Algae | 23 | Antiadhesive | 40.8 | 4.7 |
| Green/brown Algae | 37 | Antiadhesive | 42.3 | 4.5 |
| Bare Substrate | 9 | Antiadhesive | 71.8 | 4.9 |
| Bare Substrate | 23 | Antiadhesive | 37.1 | 4.2 |
| Bare Substrate | 37 | Antiadhesive | 17.4 | 2.2 |
| CCA | 9 | DCOIT | 0.1 | 0 |
| CCA | 23 | DCOIT | 1.8 | 0.8 |
| CCA | 37 | DCOIT | 1.1 | 0.3 |
| Green/brown Algae | 9 | DCOIT | 38.3 | 5 |
| Green/brown Algae | 23 | DCOIT | 25.6 | 4.9 |
| Green/brown Algae | 37 | DCOIT | 36.2 | 3.4 |
| Bare Substrate | 9 | DCOIT | 61.6 | 5 |
| Bare Substrate | 23 | DCOIT | 72.5 | 5.2 |
| Bare Substrate | 37 | DCOIT | 62.8 | 3.3 |

**Table S2: Estimated marginal means (EMM), standard error (SE), degrees of freedom (df) and upper and lower confidence levels (CL) of each fouling class (CCA, green/brown algae, bare substrate) and treatment (Control, Nanoparticles, Antiadhesive, DCOIT) combination after 37 days on the fully-coated (FC) plugs.** Intervals (EMM, SE, CL) were back-transformed from the square-root scale.

| **Treatment** | **Fouling class** | **EMM** | **Standard error** | **df** | **Lower CL** | **Upper CL** |
| --- | --- | --- | --- | --- | --- | --- |
| Control | CCA | 20.4 | 4.9 | 2 | 4.8 | 47 |
| Nanoparticles | CCA | 22.4 | 5.1 | 2 | 5.8 | 49.9 |
| Antiadhesive | CCA | 31.4 | 6.1 | 2 | 10.7 | 63 |
| DCOIT | CCA | 0.6 | 0.9 | 2 | 0 | 9.7 |
| Control | Green/brown Algae | 58.2 | 7.5 | 2 | 30.5 | 94.9 |
| Nanoparticles | Green/brown Algae | 57.1 | 7.4 | 2 | 29.7 | 93.5 |
| Antiadhesive | Green/brown Algae | 35 | 5.8 | 2 | 14.5 | 64.5 |
| DCOIT | Green/brown Algae | 32.8 | 5.6 | 2 | 13.1 | 61.5 |
| Control | Bare Substrate | 3.5 | 1.4 | 2 | 0.1 | 12.3 |
| Nanoparticles | Bare Substrate | 1.6 | 1 | 2 | 0 | 8.3 |
| Antiadhesive | Bare Substrate | 14.1 | 2.8 | 2 | 4.5 | 28.9 |
| DCOIT | Bare Substrate | 60.3 | 5.9 | 2 | 37.7 | 88.1 |

**Table S3: Results of pairwise post-hoc tests of fouling coverage data after 37 days on the fully-coated (FC) plugs based on estimated marginal means. Fouling coverage was compared between treatments (Control, Nanoparticles, Antiadhesive, DCOIT), within fouling classes (CCA, green/brown algae, bare substrate).** Note that the estimated contrasts are on the square-root scale. Tests were performed on the square-root scale. The p-value was adjusted for multiple comparisons (family of 4 estimates) with the Tukey method. Significant codes indicate: * < 0.05, ** < 0.01, *** < 0.001.

| **Compared treatment pair** | **Fouling class** | **Estimated**  **contrast** | **Standard error** | **df** | **t ratio** | **p-value** |
| --- | --- | --- | --- | --- | --- | --- |
| Control - Nanoparticles | CCA | -0.2 | 0.6 | 526 | -0.347 | 0.986 |
| Control - Antiadhesive | CCA | -1.1 | 0.6 | 526 | -1.774 | 0.287 |
| Control - DCOIT | CCA | 3.7 | 0.6 | 526 | 6.127 | < 0.001 *** |
| Nanoparticles - Antiadhesive | CCA | -0.9 | 0.6 | 526 | -1.427 | 0.483 |
| Nanoparticles - DCOIT | CCA | 3.9 | 0.6 | 526 | 6.474 | < 0.001 *** |
| Antiadhesive - DCOIT | CCA | 4.8 | 0.6 | 526 | 7.901 | < 0.001 *** |
| Control - Nanoparticles | Green/brown Algae | 0.1 | 0.5 | 526 | 0.142 | 0.999 |
| Control - Antiadhesive | Green/brown Algae | 1.7 | 0.5 | 526 | 3.317 | 0.005 ** |
| Control - DCOIT | Green/brown Algae | 1.9 | 0.5 | 526 | 3.684 | 0.001 ** |
| Nanoparticles - Antiadhesive | Green/brown Algae | 1.6 | 0.5 | 526 | 3.175 | 0.009 ** |
| Nanoparticles - DCOIT | Green/brown Algae | 1.8 | 0.5 | 526 | 3.542 | 0.002 ** |
| Antiadhesive - DCOIT | Green/brown Algae | 0.2 | 0.5 | 526 | 0.367 | 0.983 |
| Control - Nanoparticles | Bare Substrate | 0.6 | 0.3 | 526 | 2.351 | 0.088 |
| Control - Antiadhesive | Bare Substrate | -1.9 | 0.3 | 526 | -7.087 | < 0.001 *** |
| Control - DCOIT | Bare Substrate | -5.9 | 0.3 | 526 | -22.318 | < 0.001 *** |
| Nanoparticles - Antiadhesive | Bare Substrate | -2.5 | 0.3 | 526 | -9.438 | < 0.001 *** |
| Nanoparticles - DCOIT | Bare Substrate | -6.5 | 0.3 | 526 | -24.669 | < 0.001 *** |
| Antiadhesive - DCOIT | Bare Substrate | -4 | 0.3 | 526 | -15.231 | < 0.001 *** |

**Table S4: Results of full pairwise post-hoc tests of fouling coverage data after 37 days on the fully-coated (FC) plugs based on estimated marginal means. Fouling coverage was compared between all treatments (Control, Nanoparticles, Antiadhesive, DCOIT) and fouling class (CCA, green/brown algae, bare substrate) combinations.** Note that the estimated contrasts are on the square-root scale. Tests were performed on the square-root scale. The p-value was adjusted for multiple comparisons (family of 12 estimates) with the Tukey method. Significant codes indicate: * < 0.05, ** < 0.01, *** < 0.001.

| **Compared treatment-fouling class pair** | **Estimated contrast** | **Standard error** | **df** | **t ratio** | **p-value** |
| --- | --- | --- | --- | --- | --- |
| Control CCA - Nanoparticles CCA | -0.2 | 0.6 | 526 | -0.347 | 1 |
| Control CCA - Antiadhesive CCA | -1.1 | 0.6 | 526 | -1.774 | 0.831 |
| Control CCA - DCOIT CCA | 3.7 | 0.6 | 526 | 6.127 | < 0.001 *** |
| Control CCA - Control Green/brown Algae | -3.1 | 0.6 | 526 | -5.504 | < 0.001 *** |
| Control CCA - Nanoparticles Green/brown Algae | -3 | 0.6 | 526 | -5.375 | < 0.001 *** |
| Control CCA - Antiadhesive Green/brown Algae | -1.4 | 0.6 | 526 | -2.474 | 0.359 |
| Control CCA - DCOIT Green/brown Algae | -1.2 | 0.6 | 526 | -2.139 | 0.595 |
| Control CCA - Control Bare Substrate | 2.6 | 0.5 | 526 | 5.617 | < 0.001 *** |
| Control CCA - Nanoparticles Bare Substrate | 3.3 | 0.5 | 526 | 6.935 | < 0.001 *** |
| Control CCA - Antiadhesive Bare Substrate | 0.8 | 0.5 | 526 | 1.643 | 0.892 |
| Control CCA - DCOIT Bare Substrate | -3.2 | 0.5 | 526 | -6.899 | < 0.001 *** |
| Nanoparticles CCA - Antiadhesive CCA | -0.9 | 0.6 | 526 | -1.427 | 0.958 |
| Nanoparticles CCA - DCOIT CCA | 3.9 | 0.6 | 526 | 6.474 | < 0.001 *** |
| Nanoparticles CCA - Control Green/brown Algae | -2.9 | 0.6 | 526 | -5.13 | < 0.001 *** |
| Nanoparticles CCA - Nanoparticles Green/brown Algae | -2.8 | 0.6 | 526 | -5 | < 0.001 *** |
| Nanoparticles CCA - Antiadhesive Green/brown Algae | -1.2 | 0.6 | 526 | -2.099 | 0.624 |
| Nanoparticles CCA - DCOIT Green/brown Algae | -1 | 0.6 | 526 | -1.764 | 0.837 |
| Nanoparticles CCA - Control Bare Substrate | 2.9 | 0.5 | 526 | 6.067 | < 0.001 *** |
| Nanoparticles CCA - Nanoparticles Bare Substrate | 3.5 | 0.5 | 526 | 7.386 | < 0.001 *** |
| Nanoparticles CCA - Antiadhesive Bare Substrate | 1 | 0.5 | 526 | 2.094 | 0.628 |
| Nanoparticles CCA - DCOIT Bare Substrate | -3 | 0.5 | 526 | -6.448 | < 0.001 *** |
| Antiadhesive CCA - DCOIT CCA | 4.8 | 0.6 | 526 | 7.901 | < 0.001 *** |
| Antiadhesive CCA - Control Green/brown Algae | -2 | 0.6 | 526 | -3.589 | 0.019 * |
| Antiadhesive CCA - Nanoparticles Green/brown Algae | -2 | 0.6 | 526 | -3.46 | 0.029 * |
| Antiadhesive CCA - Antiadhesive Green/brown Algae | -0.3 | 0.6 | 526 | -0.559 | 1 |
| Antiadhesive CCA - DCOIT Green/brown Algae | -0.1 | 0.6 | 526 | -0.223 | 1 |
| Antiadhesive CCA - Control Bare Substrate | 3.7 | 0.5 | 526 | 7.92 | < 0.001 *** |
| Antiadhesive CCA - Nanoparticles Bare Substrate | 4.3 | 0.5 | 526 | 9.239 | < 0.001 *** |
| Antiadhesive CCA - Antiadhesive Bare Substrate | 1.9 | 0.5 | 526 | 3.946 | 0.005 ** |
| Antiadhesive CCA - DCOIT Bare Substrate | -2.2 | 0.5 | 526 | -4.595 | < 0.001 *** |
| DCOIT CCA - Control Green/brown Algae | -6.8 | 0.6 | 526 | -12.118 | < 0.001 *** |
| DCOIT CCA - Nanoparticles Green/brown Algae | -6.8 | 0.6 | 526 | -11.989 | < 0.001 *** |
| DCOIT CCA - Antiadhesive Green/brown Algae | -5.1 | 0.6 | 526 | -9.088 | < 0.001 *** |
| DCOIT CCA - DCOIT Green/brown Algae | -4.9 | 0.6 | 526 | -8.752 | < 0.001 *** |
| DCOIT CCA - Control Bare Substrate | -1.1 | 0.5 | 526 | -2.337 | 0.451 |
| DCOIT CCA - Nanoparticles Bare Substrate | -0.5 | 0.5 | 526 | -1.019 | 0.997 |
| DCOIT CCA - Antiadhesive Bare Substrate | -3 | 0.5 | 526 | -6.311 | < 0.001 *** |
| DCOIT CCA - DCOIT Bare Substrate | -7 | 0.5 | 526 | -14.853 | < 0.001 *** |
| Control Green & Brown Algae - Nanoparticles Green/brown Algae | 0.1 | 0.5 | 526 | 0.142 | 1 |
| Control Green & Brown Algae - Antiadhesive Green/brown Algae | 1.7 | 0.5 | 526 | 3.317 | 0.045 * |
| Control Green & Brown Algae - DCOIT Green/brown Algae | 1.9 | 0.5 | 526 | 3.684 | 0.013 * |
| Control Green & Brown Algae - Control Bare Substrate | 5.7 | 0.4 | 526 | 14.028 | < 0.001 *** |
| Control Green & Brown Algae - Nanoparticles Bare Substrate | 6.4 | 0.4 | 526 | 15.54 | < 0.001 *** |
| Control Green & Brown Algae - Antiadhesive Bare Substrate | 3.9 | 0.4 | 526 | 9.472 | < 0.001 *** |
| Control Green & Brown Algae - DCOIT Bare Substrate | -0.1 | 0.4 | 526 | -0.319 | 1 |
| Nanoparticles Green/brown Algae - Antiadhesive Green/brown Algae | 1.6 | 0.5 | 526 | 3.175 | 0.069 |
| Nanoparticles Green/brown Algae - DCOIT Green/brown Algae | 1.8 | 0.5 | 526 | 3.542 | 0.022 * |
| Nanoparticles Green/brown Algae - Control Bare Substrate | 5.7 | 0.4 | 526 | 13.85 | < 0.001 *** |
| Nanoparticles Green/brown Algae - Nanoparticles Bare Substrate | 6.3 | 0.4 | 526 | 15.361 | < 0.001 *** |
| Nanoparticles Green/brown Algae - Antiadhesive Bare Substrate | 3.8 | 0.4 | 526 | 9.294 | < 0.001 *** |
| Nanoparticles Green/brown Algae - DCOIT Bare Substrate | -0.2 | 0.4 | 526 | -0.498 | 1 |
| Antiadhesive Green/brown Algae - DCOIT Green/brown Algae | 0.2 | 0.5 | 526 | 0.367 | 1 |
| Antiadhesive Green/brown Algae - Control Bare Substrate | 4 | 0.4 | 526 | 9.85 | < 0.001 *** |
| Antiadhesive Green/brown Algae - Nanoparticles Bare Substrate | 4.7 | 0.4 | 526 | 11.362 | < 0.001 *** |
| Antiadhesive Green/brown Algae - Antiadhesive Bare Substrate | 2.2 | 0.4 | 526 | 5.295 | < 0.001 *** |
| Antiadhesive Green/brown Algae - DCOIT Bare Substrate | -1.8 | 0.4 | 526 | -4.497 | < 0.001 *** |
| DCOIT Green/brown Algae - Control Bare Substrate | 3.8 | 0.4 | 526 | 9.388 | < 0.001 *** |
| DCOIT Green/brown Algae - Nanoparticles Bare Substrate | 4.5 | 0.4 | 526 | 10.899 | < 0.001 *** |
| DCOIT Green/brown Algae - Antiadhesive Bare Substrate | 2 | 0.4 | 526 | 4.832 | < 0.001 *** |
| DCOIT Green/brown Algae - DCOIT Bare Substrate | -2 | 0.4 | 526 | -4.96 | < 0.001 *** |
| Control Bare Substrate - Nanoparticles Bare Substrate | 0.6 | 0.3 | 526 | 2.351 | 0.441 |
| Control Bare Substrate - Antiadhesive Bare Substrate | -1.9 | 0.3 | 526 | -7.087 | < 0.001 *** |
| Control Bare Substrate - DCOIT Bare Substrate | -5.9 | 0.3 | 526 | -22.318 | < 0.001 *** |
| Nanoparticles Bare Substrate - Antiadhesive Bare Substrate | -2.5 | 0.3 | 526 | -9.438 | < 0.001 *** |
| Nanoparticles Bare Substrate - DCOIT Bare Substrate | -6.5 | 0.3 | 526 | -24.669 | < 0.001 *** |
| Antiadhesive Bare Substrate - DCOIT Bare Substrate | -4 | 0.3 | 526 | -15.231 | < 0.001 *** |

**Table S5: Summary statistics of fouling coverage data for each fouling class (CCA, green/brown algae, bare substrate), treatment (Nanoparticles, Antiadhesive, DCOIT) and area (coated/uncoated) combination at each monitoring period (9, 23, 37 days) on the partially-coated (PC) plugs.** Values represent mean percent fouling coverage (%) per area. N (= 45) corresponds to the number of plugs in each treatment measured repeatedly for each monitoring period.

| **Fouling class** | **Days** | **Treatment** | **Area** | **Mean fouling coverage** | **Standard error** |
| --- | --- | --- | --- | --- | --- |
| CCA | 9 | Nanoparticles | Coated | 0.4 | 0.1 |
| CCA | 9 | Nanoparticles | Uncoated | 0.4 | 0.1 |
| CCA | 23 | Nanoparticles | Coated | 24.1 | 4.3 |
| CCA | 23 | Nanoparticles | Uncoated | 27.2 | 4.4 |
| CCA | 37 | Nanoparticles | Coated | 34.1 | 5.6 |
| CCA | 37 | Nanoparticles | Uncoated | 36.1 | 5.7 |
| Green/brown Algae | 9 | Nanoparticles | Coated | 60.8 | 5.7 |
| Green/brown Algae | 9 | Nanoparticles | Uncoated | 51.5 | 5.4 |
| Green/brown Algae | 23 | Nanoparticles | Coated | 68 | 4.8 |
| Green/brown Algae | 23 | Nanoparticles | Uncoated | 62.5 | 5.3 |
| Green/brown Algae | 37 | Nanoparticles | Coated | 63.6 | 5.5 |
| Green/brown Algae | 37 | Nanoparticles | Uncoated | 59 | 5.5 |
| Bare Substrate | 9 | Nanoparticles | Coated | 38.9 | 5.6 |
| Bare Substrate | 9 | Nanoparticles | Uncoated | 48.1 | 5.4 |
| Bare Substrate | 23 | Nanoparticles | Coated | 7.9 | 2.4 |
| Bare Substrate | 23 | Nanoparticles | Uncoated | 10.3 | 2.5 |
| Bare Substrate | 37 | Nanoparticles | Coated | 2.3 | 0.5 |
| Bare Substrate | 37 | Nanoparticles | Uncoated | 4.9 | 1.1 |
| CCA | 9 | Antiadhesive | Coated | 0.7 | 0.1 |
| CCA | 9 | Antiadhesive | Uncoated | 0.8 | 0.2 |
| CCA | 23 | Antiadhesive | Coated | 29.3 | 4.6 |
| CCA | 23 | Antiadhesive | Uncoated | 29.5 | 4.6 |
| CCA | 37 | Antiadhesive | Coated | 43.9 | 5.5 |
| CCA | 37 | Antiadhesive | Uncoated | 37.4 | 5.4 |
| Green/brown Algae | 9 | Antiadhesive | Coated | 30.5 | 5.5 |
| Green/brown Algae | 9 | Antiadhesive | Uncoated | 42.8 | 5 |
| Green/brown Algae | 23 | Antiadhesive | Coated | 49 | 4.9 |
| Green/brown Algae | 23 | Antiadhesive | Uncoated | 57.6 | 5 |
| Green/brown Algae | 37 | Antiadhesive | Coated | 45.1 | 5 |
| Green/brown Algae | 37 | Antiadhesive | Uncoated | 56.5 | 5.2 |
| Bare Substrate | 9 | Antiadhesive | Coated | 68.8 | 5.5 |
| Bare Substrate | 9 | Antiadhesive | Uncoated | 56.5 | 5 |
| Bare Substrate | 23 | Antiadhesive | Coated | 21.7 | 2.8 |
| Bare Substrate | 23 | Antiadhesive | Uncoated | 12.9 | 1.6 |
| Bare Substrate | 37 | Antiadhesive | Coated | 11 | 1.8 |
| Bare Substrate | 37 | Antiadhesive | Uncoated | 6.1 | 0.9 |
| CCA | 9 | DCOIT | Coated | 0.1 | 0 |
| CCA | 9 | DCOIT | Uncoated | 0.2 | 0 |
| CCA | 23 | DCOIT | Coated | 2.9 | 1.6 |
| CCA | 23 | DCOIT | Uncoated | 22.4 | 3.4 |
| CCA | 37 | DCOIT | Coated | 5.2 | 1.3 |
| CCA | 37 | DCOIT | Uncoated | 22.5 | 4 |
| Green/brown Algae | 9 | DCOIT | Coated | 33.3 | 4.6 |
| Green/brown Algae | 9 | DCOIT | Uncoated | 62 | 5.8 |
| Green/brown Algae | 23 | DCOIT | Coated | 30.6 | 4.6 |
| Green/brown Algae | 23 | DCOIT | Uncoated | 66.5 | 3.6 |
| Green/brown Algae | 37 | DCOIT | Coated | 33.9 | 3.3 |
| Green/brown Algae | 37 | DCOIT | Uncoated | 69.4 | 3.9 |
| Bare Substrate | 9 | DCOIT | Coated | 66.5 | 4.6 |
| Bare Substrate | 9 | DCOIT | Uncoated | 37.8 | 5.8 |
| Bare Substrate | 23 | DCOIT | Coated | 66.5 | 4.9 |
| Bare Substrate | 23 | DCOIT | Uncoated | 11.1 | 1.5 |
| Bare Substrate | 37 | DCOIT | Coated | 60.9 | 3.3 |
| Bare Substrate | 37 | DCOIT | Uncoated | 8.1 | 1.2 |

**Table S6: Estimated marginal means (EMM), standard error (SE), degrees of freedom (df) and upper and lower confidence levels (CL) of each area (coated/uncoated), treatment (Nanoparticles, Antiadhesive, DCOIT) and fouling class (CCA, green/brown algae, bare substrate) combination after 37 days on the areas (coated vs. uncoated) of the partially-coated (PC) plugs.** Intervals (EMM, SE, CL) were back-transformed from the square-root scale.

| **Area** | **Treatment** | **Fouling class** | **EMM** | **Standard error** | **df** | **Lower CL** | **Upper CL** |
| --- | --- | --- | --- | --- | --- | --- | --- |
| Coated | Nanoparticles | CCA | 23.7 | 6.5 | 2 | 4 | 59.7 |
| Uncoated | Nanoparticles | CCA | 24.1 | 6.5 | 2 | 4.2 | 60.4 |
| Coated | Antiadhesive | CCA | 35 | 7.9 | 2 | 9.3 | 77 |
| Uncoated | Antiadhesive | CCA | 27 | 6.9 | 2 | 5.5 | 65 |
| Coated | DCOIT | CCA | 3.1 | 2.4 | 2 | 0 | 21.5 |
| Uncoated | DCOIT | CCA | 14 | 5 | 2 | 0.8 | 43.5 |
| Coated | Nanoparticles | Green/brown Algae | 55.6 | 8.5 | 2 | 25 | 98.3 |
| Uncoated | Nanoparticles | Green/brown Algae | 50 | 8.1 | 2 | 21.3 | 90.8 |
| Coated | Antiadhesive | Green/brown Algae | 36.7 | 6.9 | 2 | 13 | 72.6 |
| Uncoated | Antiadhesive | Green/brown Algae | 48.8 | 8 | 2 | 20.5 | 89.2 |
| Coated | DCOIT | Green/brown Algae | 30.8 | 6.3 | 2 | 9.6 | 64.2 |
| Uncoated | DCOIT | Green/brown Algae | 66.3 | 9.3 | 2 | 32.3 | 112.4 |
| Coated | Nanoparticles | Bare Substrate | 1.5 | 1.2 | 2 | 0 | 10.8 |
| Uncoated | Nanoparticles | Bare Substrate | 2.9 | 1.6 | 2 | 0 | 14.3 |
| Coated | Antiadhesive | Bare Substrate | 8.1 | 2.7 | 2 | 0.6 | 24.1 |
| Uncoated | Antiadhesive | Bare Substrate | 4.7 | 2.1 | 2 | 0 | 18 |
| Coated | DCOIT | Bare Substrate | 58.1 | 7.3 | 2 | 30.8 | 94 |
| Uncoated | DCOIT | Bare Substrate | 6.3 | 2.4 | 2 | 0.2 | 21 |

**Table S7: Results of pairwise post-hoc tests of fouling coverage data (CCA, green/brown algae, bare substrate) after 37 days on the partially-coated (PC) plugs based on estimated marginal means. Fouling coverage was compared between areas (coated/uncoated), within treatment (Nanoparticles, Antiadhesive, DCOIT) and fouling class (CCA, green/brown algae, bare substrate).** Note that the estimated contrasts are on the square-root scale. Tests were performed on the square-root scale. Significant codes indicate: * < 0.05, ** < 0.01, *** < 0.001.

| **Compared treatment-area pair** | **Fouling class** | **Estimated contrast** | **Standard error** | **df** | **t ratio** | **p-value** |
| --- | --- | --- | --- | --- | --- | --- |
| Nanoparticles: Coated - Uncoated | CCA | 0 | 0.7 | 790 | -0.064 | 0.949 |
| Antiadhesive: Coated - Uncoated | CCA | 0.7 | 0.7 | 790 | 1.029 | 0.304 |
| DCOIT: Coated - Uncoated | CCA | -2 | 0.7 | 790 | -2.828 | 0.005 ** |
| Nanoparticles: Coated - Uncoated | Green/brown Algae | 0.4 | 0.5 | 790 | 0.779 | 0.436 |
| Antiadhesive: Coated - Uncoated | Green/brown Algae | -0.9 | 0.5 | 790 | -1.844 | 0.066 |
| DCOIT: Coated - Uncoated | Green/brown Algae | -2.6 | 0.5 | 790 | -5.167 | < 0.001 *** |
| Nanoparticles: Coated - Uncoated | Bare Substrate | -0.5 | 0.3 | 790 | -1.968 | 0.049 * |
| Antiadhesive: Coated - Uncoated | Bare Substrate | 0.7 | 0.3 | 790 | 2.647 | 0.008 ** |
| DCOIT: Coated - Uncoated | Bare Substrate | 5.1 | 0.3 | 790 | 20.395 | < 0.001 *** |

**Table S8: Results of full pairwise post-hoc tests of fouling coverage data after 37 days on the partially-coated (PC) plugs based on estimated marginal means. Fouling coverage was compared between all area (coated/uncoated), treatment (Nanoparticles, Antiadhesive, DCOIT) and fouling class (CCA, green/brown algae, bare substrate) combinations.** Note that the estimated contrasts are on the square-root scale. Tests were performed on the square-root scale. The p-value was adjusted for multiple comparisons (family of 18 estimates) with the Tukey method. Significant codes indicate: * < 0.05, ** < 0.01, *** < 0.001.

| **Compared area-treatment-fouling class pair** | **Estimated contrast** | **Standard error** | **df** | **t ratio** | **p-value** |
| --- | --- | --- | --- | --- | --- |
| Coated Nanoparticles CCA - Uncoated Nanoparticles CCA | 0 | 0.7 | 790 | -0.064 | 1 |
| Coated Nanoparticles CCA - Coated Antiadhesive CCA | -1.1 | 0.7 | 790 | -1.514 | 0.99 |
| Coated Nanoparticles CCA - Uncoated Antiadhesive CCA | -0.3 | 0.7 | 790 | -0.485 | 1 |
| Coated Nanoparticles CCA - Coated DCOIT CCA | 3.1 | 0.7 | 790 | 4.447 | 0.001 ** |
| Coated Nanoparticles CCA - Uncoated DCOIT CCA | 1.1 | 0.7 | 790 | 1.62 | 0.979 |
| Coated Nanoparticles CCA - Coated Nanoparticles Green/brown Algae | -2.6 | 0.6 | 790 | -4.284 | 0.003 ** |
| Coated Nanoparticles CCA - Uncoated Nanoparticles Green/brown Algae | -2.2 | 0.6 | 790 | -3.64 | 0.032 * |
| Coated Nanoparticles CCA - Coated Antiadhesive Green/brown Algae | -1.2 | 0.6 | 790 | -1.976 | 0.88 |
| Coated Nanoparticles CCA - Uncoated Antiadhesive Green/brown Algae | -2.1 | 0.6 | 790 | -3.502 | 0.05 |
| Coated Nanoparticles CCA - Coated DCOIT Green/brown Algae | -0.7 | 0.6 | 790 | -1.138 | 1 |
| Coated Nanoparticles CCA - Uncoated DCOIT Green/brown Algae | -3.3 | 0.6 | 790 | -5.413 | < 0.001 *** |
| Coated Nanoparticles CCA - Coated Nanoparticles Bare Substrate | 3.6 | 0.5 | 790 | 6.98 | < 0.001 *** |
| Coated Nanoparticles CCA - Uncoated Nanoparticles Bare Substrate | 3.2 | 0.5 | 790 | 6.036 | < 0.001 *** |
| Coated Nanoparticles CCA - Coated Antiadhesive Bare Substrate | 2 | 0.5 | 790 | 3.878 | 0.014 * |
| Coated Nanoparticles CCA - Uncoated Antiadhesive Bare Substrate | 2.7 | 0.5 | 790 | 5.148 | < 0.001 *** |
| Coated Nanoparticles CCA - Coated DCOIT Bare Substrate | -2.8 | 0.5 | 790 | -5.285 | < 0.001 *** |
| Coated Nanoparticles CCA - Uncoated DCOIT Bare Substrate | 2.3 | 0.5 | 790 | 4.498 | 0.001 ** |
| Uncoated Nanoparticles CCA - Coated Antiadhesive CCA | -1 | 0.7 | 790 | -1.449 | 0.994 |
| Uncoated Nanoparticles CCA - Uncoated Antiadhesive CCA | -0.3 | 0.7 | 790 | -0.421 | 1 |
| Uncoated Nanoparticles CCA - Coated DCOIT CCA | 3.1 | 0.7 | 790 | 4.512 | 0.001 ** |
| Uncoated Nanoparticles CCA - Uncoated DCOIT CCA | 1.2 | 0.7 | 790 | 1.684 | 0.969 |
| Uncoated Nanoparticles CCA - Coated Nanoparticles Green/brown Algae | -2.6 | 0.6 | 790 | -4.21 | 0.004 ** |
| Uncoated Nanoparticles CCA - Uncoated Nanoparticles Green/brown Algae | -2.2 | 0.6 | 790 | -3.566 | 0.04 * |
| Uncoated Nanoparticles CCA - Coated Antiadhesive Green/brown Algae | -1.2 | 0.6 | 790 | -1.902 | 0.911 |
| Uncoated Nanoparticles CCA - Uncoated Antiadhesive Green/brown Algae | -2.1 | 0.6 | 790 | -3.428 | 0.063 |
| Uncoated Nanoparticles CCA - Coated DCOIT Green/brown Algae | -0.6 | 0.6 | 790 | -1.064 | 1 |
| Uncoated Nanoparticles CCA - Uncoated DCOIT Green/brown Algae | -3.2 | 0.6 | 790 | -5.339 | < 0.001 *** |
| Uncoated Nanoparticles CCA - Coated Nanoparticles Bare Substrate | 3.7 | 0.5 | 790 | 7.066 | < 0.001 *** |
| Uncoated Nanoparticles CCA - Uncoated Nanoparticles Bare Substrate | 3.2 | 0.5 | 790 | 6.122 | < 0.001 *** |
| Uncoated Nanoparticles CCA - Coated Antiadhesive Bare Substrate | 2.1 | 0.5 | 790 | 3.964 | 0.01 |
| Uncoated Nanoparticles CCA - Uncoated Antiadhesive Bare Substrate | 2.7 | 0.5 | 790 | 5.234 | < 0.001 *** |
| Uncoated Nanoparticles CCA - Coated DCOIT Bare Substrate | -2.7 | 0.5 | 790 | -5.199 | < 0.001 *** |
| Uncoated Nanoparticles CCA - Uncoated DCOIT Bare Substrate | 2.4 | 0.5 | 790 | 4.584 | < 0.001 *** |
| Coated Antiadhesive CCA - Uncoated Antiadhesive CCA | 0.7 | 0.7 | 790 | 1.029 | 1 |
| Coated Antiadhesive CCA - Coated DCOIT CCA | 4.1 | 0.7 | 790 | 5.961 | < 0.001 *** |
| Coated Antiadhesive CCA - Uncoated DCOIT CCA | 2.2 | 0.7 | 790 | 3.133 | 0.145 |
| Coated Antiadhesive CCA - Coated Nanoparticles Green/brown Algae | -1.5 | 0.6 | 790 | -2.548 | 0.494 |
| Coated Antiadhesive CCA - Uncoated Nanoparticles Green/brown Algae | -1.2 | 0.6 | 790 | -1.904 | 0.91 |
| Coated Antiadhesive CCA - Coated Antiadhesive Green/brown Algae | -0.1 | 0.6 | 790 | -0.24 | 1 |
| Coated Antiadhesive CCA - Uncoated Antiadhesive Green/brown Algae | -1.1 | 0.6 | 790 | -1.766 | 0.952 |
| Coated Antiadhesive CCA - Coated DCOIT Green/brown Algae | 0.4 | 0.6 | 790 | 0.598 | 1 |
| Coated Antiadhesive CCA - Uncoated DCOIT Green/brown Algae | -2.2 | 0.6 | 790 | -3.677 | 0.028 * |
| Coated Antiadhesive CCA - Coated Nanoparticles Bare Substrate | 4.7 | 0.5 | 790 | 8.994 | < 0.001 *** |
| Coated Antiadhesive CCA - Uncoated Nanoparticles Bare Substrate | 4.2 | 0.5 | 790 | 8.05 | < 0.001 *** |
| Coated Antiadhesive CCA - Coated Antiadhesive Bare Substrate | 3.1 | 0.5 | 790 | 5.892 | < 0.001 *** |
| Coated Antiadhesive CCA - Uncoated Antiadhesive Bare Substrate | 3.7 | 0.5 | 790 | 7.162 | < 0.001 *** |
| Coated Antiadhesive CCA - Coated DCOIT Bare Substrate | -1.7 | 0.5 | 790 | -3.271 | 0.1 |
| Coated Antiadhesive CCA - Uncoated DCOIT Bare Substrate | 3.4 | 0.5 | 790 | 6.512 | < 0.001 *** |
| Uncoated Antiadhesive CCA - Coated DCOIT CCA | 3.4 | 0.7 | 790 | 4.932 | < 0.001 *** |
| Uncoated Antiadhesive CCA - Uncoated DCOIT CCA | 1.5 | 0.7 | 790 | 2.105 | 0.813 |
| Uncoated Antiadhesive CCA - Coated Nanoparticles Green/brown Algae | -2.3 | 0.6 | 790 | -3.728 | 0.023 * |
| Uncoated Antiadhesive CCA - Uncoated Nanoparticles Green/brown Algae | -1.9 | 0.6 | 790 | -3.084 | 0.165 |
| Uncoated Antiadhesive CCA - Coated Antiadhesive Green/brown Algae | -0.9 | 0.6 | 790 | -1.419 | 0.995 |
| Uncoated Antiadhesive CCA - Uncoated Antiadhesive Green/brown Algae | -1.8 | 0.6 | 790 | -2.946 | 0.23 |
| Uncoated Antiadhesive CCA - Coated DCOIT Green/brown Algae | -0.4 | 0.6 | 790 | -0.581 | 1 |
| Uncoated Antiadhesive CCA - Uncoated DCOIT Green/brown Algae | -2.9 | 0.6 | 790 | -4.857 | < 0.001 *** |
| Uncoated Antiadhesive CCA - Coated Nanoparticles Bare Substrate | 4 | 0.5 | 790 | 7.625 | < 0.001 *** |
| Uncoated Antiadhesive CCA - Uncoated Nanoparticles Bare Substrate | 3.5 | 0.5 | 790 | 6.682 | < 0.001 *** |
| Uncoated Antiadhesive CCA - Coated Antiadhesive Bare Substrate | 2.4 | 0.5 | 790 | 4.524 | < 0.001 *** |
| Uncoated Antiadhesive CCA - Uncoated Antiadhesive Bare Substrate | 3 | 0.5 | 790 | 5.793 | < 0.001 *** |
| Uncoated Antiadhesive CCA - Coated DCOIT Bare Substrate | -2.4 | 0.5 | 790 | -4.64 | < 0.001 *** |
| Uncoated Antiadhesive CCA - Uncoated DCOIT Bare Substrate | 2.7 | 0.5 | 790 | 5.143 | < 0.001 *** |
| Coated DCOIT CCA - Uncoated DCOIT CCA | -2 | 0.7 | 790 | -2.828 | 0.298 |
| Coated DCOIT CCA - Coated Nanoparticles Green/brown Algae | -5.7 | 0.6 | 790 | -9.384 | < 0.001 *** |
| Coated DCOIT CCA - Uncoated Nanoparticles Green/brown Algae | -5.3 | 0.6 | 790 | -8.74 | < 0.001 *** |
| Coated DCOIT CCA - Coated Antiadhesive Green/brown Algae | -4.3 | 0.6 | 790 | -7.076 | < 0.001 *** |
| Coated DCOIT CCA - Uncoated Antiadhesive Green/brown Algae | -5.2 | 0.6 | 790 | -8.602 | < 0.001 *** |
| Coated DCOIT CCA - Coated DCOIT Green/brown Algae | -3.8 | 0.6 | 790 | -6.238 | < 0.001 *** |
| Coated DCOIT CCA - Uncoated DCOIT Green/brown Algae | -6.4 | 0.6 | 790 | -10.514 | < 0.001 *** |
| Coated DCOIT CCA - Coated Nanoparticles Bare Substrate | 0.6 | 0.5 | 790 | 1.064 | 1 |
| Coated DCOIT CCA - Uncoated Nanoparticles Bare Substrate | 0.1 | 0.5 | 790 | 0.12 | 1 |
| Coated DCOIT CCA - Coated Antiadhesive Bare Substrate | -1.1 | 0.5 | 790 | -2.038 | 0.85 |
| Coated DCOIT CCA - Uncoated Antiadhesive Bare Substrate | -0.4 | 0.5 | 790 | -0.768 | 1 |
| Coated DCOIT CCA - Coated DCOIT Bare Substrate | -5.9 | 0.5 | 790 | -11.201 | < 0.001 *** |
| Coated DCOIT CCA - Uncoated DCOIT Bare Substrate | -0.7 | 0.5 | 790 | -1.419 | 0.995 |
| Uncoated DCOIT CCA - Coated Nanoparticles Green/brown Algae | -3.7 | 0.6 | 790 | -6.142 | < 0.001 *** |
| Uncoated DCOIT CCA - Uncoated Nanoparticles Green/brown Algae | -3.3 | 0.6 | 790 | -5.497 | < 0.001 *** |
| Uncoated DCOIT CCA - Coated Antiadhesive Green/brown Algae | -2.3 | 0.6 | 790 | -3.833 | 0.016 * |
| Uncoated DCOIT CCA - Uncoated Antiadhesive Green/brown Algae | -3.2 | 0.6 | 790 | -5.359 | < 0.001 *** |
| Uncoated DCOIT CCA - Coated DCOIT Green/brown Algae | -1.8 | 0.6 | 790 | -2.995 | 0.205 |
| Uncoated DCOIT CCA - Uncoated DCOIT Green/brown Algae | -4.4 | 0.6 | 790 | -7.271 | < 0.001 *** |
| Uncoated DCOIT CCA - Coated Nanoparticles Bare Substrate | 2.5 | 0.5 | 790 | 4.825 | < 0.001 *** |
| Uncoated DCOIT CCA - Uncoated Nanoparticles Bare Substrate | 2 | 0.5 | 790 | 3.882 | 0.013 * |
| Uncoated DCOIT CCA - Coated Antiadhesive Bare Substrate | 0.9 | 0.5 | 790 | 1.724 | 0.962 |
| Uncoated DCOIT CCA - Uncoated Antiadhesive Bare Substrate | 1.6 | 0.5 | 790 | 2.994 | 0.206 |
| Uncoated DCOIT CCA - Coated DCOIT Bare Substrate | -3.9 | 0.5 | 790 | -7.439 | < 0.001 *** |
| Uncoated DCOIT CCA - Uncoated DCOIT Bare Substrate | 1.2 | 0.5 | 790 | 2.343 | 0.651 |
| Coated Nanoparticles Green/brown Algae - Uncoated Nanoparticles Green/brown Algae | 0.4 | 0.5 | 790 | 0.779 | 1 |
| Coated Nanoparticles Green/brown Algae - Coated Antiadhesive Green/brown Algae | 1.4 | 0.5 | 790 | 2.79 | 0.321 |
| Coated Nanoparticles Green/brown Algae - Uncoated Antiadhesive Green/brown Algae | 0.5 | 0.5 | 790 | 0.946 | 1 |
| Coated Nanoparticles Green/brown Algae - Coated DCOIT Green/brown Algae | 1.9 | 0.5 | 790 | 3.803 | 0.018 * |
| Coated Nanoparticles Green/brown Algae - Uncoated DCOIT Green/brown Algae | -0.7 | 0.5 | 790 | -1.364 | 0.997 |
| Coated Nanoparticles Green/brown Algae - Coated Nanoparticles Bare Substrate | 6.2 | 0.4 | 790 | 15.749 | < 0.001 *** |
| Coated Nanoparticles Green/brown Algae - Uncoated Nanoparticles Bare Substrate | 5.7 | 0.4 | 790 | 14.505 | < 0.001 *** |
| Coated Nanoparticles Green/brown Algae - Coated Antiadhesive Bare Substrate | 4.6 | 0.4 | 790 | 11.662 | < 0.001 *** |
| Coated Nanoparticles Green/brown Algae - Uncoated Antiadhesive Bare Substrate | 5.3 | 0.4 | 790 | 13.335 | < 0.001 *** |
| Coated Nanoparticles Green/brown Algae - Coated DCOIT Bare Substrate | -0.2 | 0.4 | 790 | -0.415 | 1 |
| Coated Nanoparticles Green/brown Algae - Uncoated DCOIT Bare Substrate | 4.9 | 0.4 | 790 | 12.478 | < 0.001 *** |
| Uncoated Nanoparticles Green/brown Algae - Coated Antiadhesive Green/brown Algae | 1 | 0.5 | 790 | 2.011 | 0.863 |
| Uncoated Nanoparticles Green/brown Algae - Uncoated Antiadhesive Green/brown Algae | 0.1 | 0.5 | 790 | 0.167 | 1 |
| Uncoated Nanoparticles Green/brown Algae - Coated DCOIT Green/brown Algae | 1.5 | 0.5 | 790 | 3.024 | 0.191 |
| Uncoated Nanoparticles Green/brown Algae - Uncoated DCOIT Green/brown Algae | -1.1 | 0.5 | 790 | -2.143 | 0.79 |
| Uncoated Nanoparticles Green/brown Algae - Coated Nanoparticles Bare Substrate | 5.9 | 0.4 | 790 | 14.764 | < 0.001 *** |
| Uncoated Nanoparticles Green/brown Algae - Uncoated Nanoparticles Bare Substrate | 5.4 | 0.4 | 790 | 13.521 | < 0.001 *** |
| Uncoated Nanoparticles Green/brown Algae - Coated Antiadhesive Bare Substrate | 4.2 | 0.4 | 790 | 10.677 | < 0.001 *** |
| Uncoated Nanoparticles Green/brown Algae - Uncoated Antiadhesive Bare Substrate | 4.9 | 0.4 | 790 | 12.35 | < 0.001 *** |
| Uncoated Nanoparticles Green/brown Algae - Coated DCOIT Bare Substrate | -0.6 | 0.4 | 790 | -1.4 | 0.996 |
| Uncoated Nanoparticles Green/brown Algae - Uncoated DCOIT Bare Substrate | 4.6 | 0.4 | 790 | 11.493 | < 0.001 *** |
| Coated Antiadhesive Green/brown Algae - Uncoated Antiadhesive Green/brown Algae | -0.9 | 0.5 | 790 | -1.844 | 0.931 |
| Coated Antiadhesive Green/brown Algae - Coated DCOIT Green/brown Algae | 0.5 | 0.5 | 790 | 1.013 | 1 |
| Coated Antiadhesive Green/brown Algae - Uncoated DCOIT Green/brown Algae | -2.1 | 0.5 | 790 | -4.154 | 0.005 ** |
| Coated Antiadhesive Green/brown Algae - Coated Nanoparticles Bare Substrate | 4.8 | 0.4 | 790 | 12.22 | < 0.001 *** |
| Coated Antiadhesive Green/brown Algae - Uncoated Nanoparticles Bare Substrate | 4.3 | 0.4 | 790 | 10.976 | < 0.001 *** |
| Coated Antiadhesive Green/brown Algae - Coated Antiadhesive Bare Substrate | 3.2 | 0.4 | 790 | 8.132 | < 0.001 *** |
| Coated Antiadhesive Green/brown Algae - Uncoated Antiadhesive Bare Substrate | 3.9 | 0.4 | 790 | 9.806 | < 0.001 *** |
| Coated Antiadhesive Green/brown Algae - Coated DCOIT Bare Substrate | -1.6 | 0.4 | 790 | -3.945 | 0.011 * |
| Coated Antiadhesive Green/brown Algae - Uncoated DCOIT Bare Substrate | 3.5 | 0.4 | 790 | 8.949 | < 0.001 *** |
| Uncoated Antiadhesive Green/brown Algae - Coated DCOIT Green/brown Algae | 1.4 | 0.5 | 790 | 2.857 | 0.28 |
| Uncoated Antiadhesive Green/brown Algae - Uncoated DCOIT Green/brown Algae | -1.2 | 0.5 | 790 | -2.31 | 0.676 |
| Uncoated Antiadhesive Green/brown Algae - Coated Nanoparticles Bare Substrate | 5.8 | 0.4 | 790 | 14.553 | < 0.001 *** |
| Uncoated Antiadhesive Green/brown Algae - Uncoated Nanoparticles Bare Substrate | 5.3 | 0.4 | 790 | 13.309 | < 0.001 *** |
| Uncoated Antiadhesive Green/brown Algae - Coated Antiadhesive Bare Substrate | 4.1 | 0.4 | 790 | 10.465 | < 0.001 *** |
| Uncoated Antiadhesive Green/brown Algae - Uncoated Antiadhesive Bare Substrate | 4.8 | 0.4 | 790 | 12.139 | < 0.001 *** |
| Uncoated Antiadhesive Green/brown Algae - Coated DCOIT Bare Substrate | -0.6 | 0.4 | 790 | -1.611 | 0.98 |
| Uncoated Antiadhesive Green/brown Algae - Uncoated DCOIT Bare Substrate | 4.5 | 0.4 | 790 | 11.282 | < 0.001 *** |
| Coated DCOIT Green/brown Algae - Uncoated DCOIT Green/brown Algae | -2.6 | 0.5 | 790 | -5.167 | < 0.001 *** |
| Coated DCOIT Green/brown Algae - Coated Nanoparticles Bare Substrate | 4.3 | 0.4 | 790 | 10.939 | < 0.001 *** |
| Coated DCOIT Green/brown Algae - Uncoated Nanoparticles Bare Substrate | 3.8 | 0.4 | 790 | 9.695 | < 0.001 *** |
| Coated DCOIT Green/brown Algae - Coated Antiadhesive Bare Substrate | 2.7 | 0.4 | 790 | 6.851 | < 0.001 *** |
| Coated DCOIT Green/brown Algae - Uncoated Antiadhesive Bare Substrate | 3.4 | 0.4 | 790 | 8.524 | < 0.001 *** |
| Coated DCOIT Green/brown Algae - Coated DCOIT Bare Substrate | -2.1 | 0.4 | 790 | -5.226 | < 0.001 *** |
| Coated DCOIT Green/brown Algae - Uncoated DCOIT Bare Substrate | 3 | 0.4 | 790 | 7.667 | < 0.001 *** |
| Uncoated DCOIT Green/brown Algae - Coated Nanoparticles Bare Substrate | 6.9 | 0.4 | 790 | 17.475 | < 0.001 *** |
| Uncoated DCOIT Green/brown Algae - Uncoated Nanoparticles Bare Substrate | 6.4 | 0.4 | 790 | 16.232 | < 0.001 *** |
| Uncoated DCOIT Green/brown Algae - Coated Antiadhesive Bare Substrate | 5.3 | 0.4 | 790 | 13.388 | < 0.001 *** |
| Uncoated DCOIT Green/brown Algae - Uncoated Antiadhesive Bare Substrate | 6 | 0.4 | 790 | 15.061 | < 0.001 *** |
| Uncoated DCOIT Green/brown Algae - Coated DCOIT Bare Substrate | 0.5 | 0.4 | 790 | 1.311 | 0.998 |
| Uncoated DCOIT Green/brown Algae - Uncoated DCOIT Bare Substrate | 5.6 | 0.4 | 790 | 14.204 | < 0.001 *** |
| Coated Nanoparticles Bare Substrate - Uncoated Nanoparticles Bare Substrate | -0.5 | 0.3 | 790 | -1.968 | 0.884 |
| Coated Nanoparticles Bare Substrate - Coated Antiadhesive Bare Substrate | -1.6 | 0.3 | 790 | -6.466 | < 0.001 *** |
| Coated Nanoparticles Bare Substrate - Uncoated Antiadhesive Bare Substrate | -1 | 0.3 | 790 | -3.819 | 0.017 * |
| Coated Nanoparticles Bare Substrate - Coated DCOIT Bare Substrate | -6.4 | 0.3 | 790 | -25.57 | < 0.001 *** |
| Coated Nanoparticles Bare Substrate - Uncoated DCOIT Bare Substrate | -1.3 | 0.3 | 790 | -5.175 | < 0.001 *** |
| Uncoated Nanoparticles Bare Substrate - Coated Antiadhesive Bare Substrate | -1.1 | 0.3 | 790 | -4.499 | 0.001 ** |
| Uncoated Nanoparticles Bare Substrate - Uncoated Antiadhesive Bare Substrate | -0.5 | 0.3 | 790 | -1.851 | 0.928 |
| Uncoated Nanoparticles Bare Substrate - Coated DCOIT Bare Substrate | -5.9 | 0.3 | 790 | -23.603 | < 0.001 *** |
| Uncoated Nanoparticles Bare Substrate - Uncoated DCOIT Bare Substrate | -0.8 | 0.3 | 790 | -3.207 | 0.119 |
| Coated Antiadhesive Bare Substrate - Uncoated Antiadhesive Bare Substrate | 0.7 | 0.3 | 790 | 2.647 | 0.42 |
| Coated Antiadhesive Bare Substrate - Coated DCOIT Bare Substrate | -4.8 | 0.3 | 790 | -19.104 | < 0.001 *** |
| Coated Antiadhesive Bare Substrate - Uncoated DCOIT Bare Substrate | 0.3 | 0.3 | 790 | 1.291 | 0.998 |
| Uncoated Antiadhesive Bare Substrate - Coated DCOIT Bare Substrate | -5.4 | 0.3 | 790 | -21.751 | < 0.001 *** |
| Uncoated Antiadhesive Bare Substrate - Uncoated DCOIT Bare Substrate | -0.3 | 0.3 | 790 | -1.356 | 0.997 |
| Coated DCOIT Bare Substrate - Uncoated DCOIT Bare Substrate | 5.1 | 0.3 | 790 | 20.395 | < 0.001 *** |

**Table S9: Summary statistics of settlement data per treatment (Control, Nanoparticles, Antiadhesive, DCOIT) on the fully-coated (FC) plugs.** Values represent total settlement and mean settlers per cm^2^. N corresponds to the number of plugs in individual jars containing 15 coral larvae per jar.

| **Treatment** | **n** | **Total settlers** | **Mean settlers/cm^2^** | **Standard deviation** | **Standard error** |
| --- | --- | --- | --- | --- | --- |
| Control | 30 | 169 | 0.8 | 0.5 | 0.1 |
| Nanoparticles | 30 | 179 | 0.8 | 0.5 | 0.1 |
| Antiadhesive | 30 | 105 | 0.5 | 0.4 | 0.1 |
| DCOIT | 30 | 120 | 0.6 | 0.5 | 0.1 |

**Table S10: Estimated marginal means (EMM), standard error (SE), degrees of freedom (df) and upper and lower confidence levels (95% and 5%, respectively) of settlers per cm^2^ in each treatment (Control, Nanoparticles, Antiadhesive, DCOIT) on the fully-coated (FC) plugs.** Intervals (EMM, SE, CL) were back-transformed from the square-root scale.

| **Treatment** | **EMM** | **Standard error** | **df** | **Lower CL** | **Upper CL** |
| --- | --- | --- | --- | --- | --- |
| Control | 0.7 | 0.2 | 114 | 0.4 | 1.1 |
| Nanoparticles | 0.8 | 0.2 | 114 | 0.4 | 1.2 |
| Antiadhesive | 0.4 | 0.1 | 114 | 0.2 | 0.7 |
| DCOIT | 0.4 | 0.1 | 114 | 0.2 | 0.7 |

**Table S11: Results of pairwise post-hoc tests of settlement data on the fully-coated (FC) plugs based on estimated marginal means. Settler density was compared between all treatment (Control, Nanoparticles, Antiadhesive, DCOIT) combinations.** The p-value was adjusted for multiple comparisons (family of 4 estimates) with the Tukey method. Note that the estimated contrasts are on the square-root scale. Tests were performed on the square-root scale. Significant codes indicate: * < 0.05, ** < 0.01, *** < 0.001.

| **Compared treatment pair** | **Estimated contrast** | **Standard error** | **df** | **t ratio** | **p-value** |
| --- | --- | --- | --- | --- | --- |
| Control - Nanoparticles | -0.1 | 0.1 | 114 | -0.604 | 0.93 |
| Control - Antiadhesive | 0.2 | 0.1 | 114 | 2.446 | 0.074 |
| Control - DCOIT | 0.2 | 0.1 | 114 | 2.463 | 0.071 |
| Nanoparticles - Antiadhesive | 0.3 | 0.1 | 114 | 3.05 | 0.015 * |
| Nanoparticles - DCOIT | 0.3 | 0.1 | 114 | 3.067 | 0.014 * |
| Antiadhesive - DCOIT | 0 | 0.1 | 114 | 0.017 | 1 |

**Table S12: Summary statistics of settlement data for each treatment (Nanoparticles, Antiadhesive, DCOIT) and area (coated/uncoated) combination on the partially-coated (PC) plugs.** Values represent total settlement and mean settler density/cm^2^ per area (coated vs. uncoated). N (= 30) corresponds to the number of plugs in individual jars containing 15 coral larvae per jar.

| **Treatment** | **Area** | **Total settlers** | **Mean settlers/cm^2^** | **Standard error** |
| --- | --- | --- | --- | --- |
| Partially-coated Nanoparticles | Coated | 164 | 0.9 | 0.1 |
| Partially-coated Nanoparticles | Uncoated | 168 | 0.9 | 0.3 |
| Partially-coated Antiadhesive | Coated | 76 | 0.4 | 0.1 |
| Partially-coated Antiadhesive | Uncoated | 176 | 0.9 | 0.3 |
| Partially-coated DCOIT | Coated | 83 | 0.4 | 0.1 |
| Partially-coated DCOIT | Uncoated | 224 | 1.2 | 0.3 |

**Table S13: Estimated marginal means (EMM), standard error (SE), degrees of freedom (df) and confidence levels (CL) of the settlers/cm^2^ for each area (coated/uncoated), within each partially-coated (PC) treatment (Nanoparticles (PN), Antiadhesive (PA), DCOIT (PD)).** Intervals were back-transformed from the square-root scale.

| **Treatment-Area** | **EMM** | **Standard error** | **df** | **Lower CL** | **Upper CL** |
| --- | --- | --- | --- | --- | --- |
| PN-Coated | 0.7 | 0.2 | 2 | 0.1 | 2.1 |
| PN-Uncoated | 0.3 | 0.2 | 2 | 0 | 1.8 |
| PA-Coated | 0.3 | 0.1 | 2 | 0 | 1.2 |
| PA-Uncoated | 0.3 | 0.2 | 2 | 0 | 1.9 |
| PD-Coated | 0.3 | 0.1 | 2 | 0 | 1.3 |
| PD-Uncoated | 0.6 | 0.3 | 2 | 0 | 2.4 |

**Table S14: Results of pairwise post-hoc tests of settlement data on the partially-coated (PC) plugs based on estimated marginal means. Settler density was compared between all area (coated/uncoated) and treatment (Nanoparticles (PN), Antiadhesive (PA), DCOIT (PD)) combinations.** Note that the estimated contrasts are on the square-root scale. Tests were performed on the square-root scale. The p-value was adjusted for multiple comparisons (family of 7 estimates) with the Tukey method. Significant codes indicate: * < 0.05, ** < 0.01, *** < 0.001.

| **Compared treatment-area pair** | **Estimated contrast** | **Standard error** | **df** | **t ratio** | **p-value** |
| --- | --- | --- | --- | --- | --- |
| PN Coated - PA Coated | 0.4 | 0.1 | 172 | 4.113 | < 0.001 *** |
| PN Coated - PD Coated | 0.3 | 0.1 | 172 | 3.69 | 0.004 ** |
| PN Coated - PN Uncoated | 0.3 | 0.2 | 172 | 2.087 | 0.299 |
| PN Coated - PA Uncoated | 0.3 | 0.2 | 172 | 1.952 | 0.374 |
| PN Coated - PD Uncoated | 0.1 | 0.2 | 172 | 0.618 | 0.99 |
| PA Coated - PD Coated | 0 | 0.1 | 172 | -0.385 | 0.999 |
| PA Coated - PN Uncoated | 0 | 0.2 | 172 | -0.123 | 1 |
| PA Coated - PA Uncoated | 0 | 0.2 | 172 | -0.285 | 1 |
| PA Coated - PD Uncoated | -0.3 | 0.2 | 172 | -1.71 | 0.527 |
| PD Coated - PN Uncoated | 0 | 0.2 | 172 | 0.085 | 1 |
| PD Coated - PA Uncoated | 0 | 0.2 | 172 | -0.074 | 1 |
| PD Coated - PD Uncoated | -0.2 | 0.2 | 172 | -1.486 | 0.674 |
| PN Uncoated - PA Uncoated | 0 | 0.2 | 172 | -0.122 | 1 |
| PN Uncoated - PD Uncoated | -0.2 | 0.2 | 172 | -1.181 | 0.845 |
| PA Uncoated - PD Uncoated | -0.2 | 0.2 | 172 | -1.064 | 0.895 |

**SI References**

1. Herget, K. *et al.* Haloperoxidase Mimicry by CeO2-x Nanorods Combats Biofouling. *Adv. Mater.* **29**, 1–8 (2017).

2. Herget, K. *et al.* Supporting Information: Haloperoxidase Mimicry by CeO2-x Nanorods Combats Biofouling. *Adv. Mater.* **29**, (2017).
